# miR675 upregulates long noncoding RNA H19 through activating EGR1 in human liver cancer

**DOI:** 10.18632/oncotarget.5579

**Published:** 2015-09-10

**Authors:** Haiyan Li, Jiao Li, Song Jia, Mengying Wu, Jiahui An, Qidi Zheng, Wei Zhang, Dongdong Lu

**Affiliations:** ^1^ School of Life Science and Technology, Tongji University, Shanghai, China; ^2^ School of Medicine, Tongji University, Shanghai, China

**Keywords:** microRNA675, lincRNA H19, early growth response protein 1, pyruvate kinase M2, hepatoma

## Abstract

microRNAs (miRNAs) are short non-coding RNAs that are involved in post-transcriptional regulation of gene expression in multicellular organisms by affecting both the stability and translation of mRNAs. miR675, embedded in H19's first exon, had been linked to the development of human cancers. Herein, we demonstrate miR675 overexpression promotes and silencing miR675 attenuated liver cancer cell growth *in vitro* and *in vivo*. Mechanistically, miR675 inhibits the heterochromatin1 isoform HP1α expression in human liver cancer cells which causes a dramatically decrease of the total histone H3 lysine 9 trimethylation (H3K9me3), histone H3 lysine 27 trimethylation (H3K27me3) and a increase of histone H3 lysine 27 acetylation(H3K27Ac). Notably, a significant reduction of the H3K9me3 and H3K27me3 and the increment of H3K27Ac occupancy on the promoter region of EGR1 triggers EGR1 transcription, translation, sumoylation and activation which upregulates lincRNA H19. Strikingly, H19 may induce and activate tumor-specific pyruvate kinase M2 (PKM2) which is essential for the Warburg effect in its dimer and for gene expression in its teramer during tumorigenesis. Our results imply that miR675 is involved in the epigenetic regulation of H3K9me3, H3k27me3 and H3K27Ac for gene expression and function during hepatocarcinogenesis (e.g. C-myc, Pim1, Ras, CyclinD1, RB1). These findings sheds light on the significance of miR675-HP1α-EGR1-H19-PKM2 cascade signaling pathway in liver cancer.

## INTRODUCTION

Primary liver cancer are an increasing global health problem, with hepatocellular carcinoma (HCC) now being the third leading cause of cancer-related mortality worldwide [1]. MicroRNAs are potent regulators of gene expression and modulate multiple cellular processes including proliferation, differentiation, apoptosis and tumorigenesis. miR675, a miRNA, embedded in H19's first exon, is expressed exclusively in the placenta. Overexpression of miR-675 in a range of embryonic and extra-embryonic cell lines results in their reduced proliferation [2]. miR675 was shown to up-regulate the essential cartilage matrix component COL2A1, and overexpression of miR-675 rescued COL2A1 levels in H19- or SOX9-depleted cells [3]. Cadherin 11 in fibroblasts and keratinocytes is a target of miR-675, and could be involved in melanogenesis through the induction of N-cadherin during epithelial-mesenchymal transition [4]. H19 regulates glioma development by deriving miR-675 which modulated Cadherin 13 expression by directly targeting the binding site within the 3′ UTR [5]. In addition, H19 maintain hematopoietic stem cell repopulating ability through a miR-675-IGFR signaling circuit [6]. However, others resports also showed the different functions of miR675. For examples, miR675 was significantly downregulated in the metastatic prostate cancer cell and directly bound with 3′UTR of transforming growth factor β induced protein (TGFBI, an extracellular matrix protein involved in cancer metastasis) mRNA to repress its translation [7]. H19 gene could inhibit human trophoblast cell proliferation via encoding miR675 that targeted NOMO1 and interferes with Nodal signaling [8]. Intriguingly, miR675 helps in discriminating adrenocortical carcinomas (ACCs) from adrenocortical adenomas (ACAs) [9]. However, the exact roles of mature miR-675 in hepatocarcinogenesis have not been identified.

Heterochromatin causes epigenetic repression that can be transmitted through multiple cell divisions. Heterochromatic-silencing factors preclude histone turnover to promote silencing and inheritance of repressive chromatin [10]. HP1α is an essential protein critical for heterochromatin assembly and regulation. HP1α nucleates with high affinity independently of H3K9me in promoters of active genes and then spreads via H3K9 methylation and transient looping contacts with those H3K9me target sites [11]. HP1 mediates the recognition and destruction of heterochromatic RNA transcripts [12]. Strikingly, HP1 promotes tumor suppressor BRCA1 functions during the DNA damage response [13]. DNA double-strand breaks promote methylation of histone H3 on lysine 9 and transient formation of repressive chromatin [14]. Recently, a research indicate the hyperacetylation of H3K9 at EGR1 binding sites in promoter region II of the GDNF gene can up-regulate the binding of EGR1 to increase GDNF gene transcription in glioma cells [15]. Arf-EGR1-C/EBPβ axis as an important determinant of cellular responses (senescence or transformation) to oncogenic Ras signaling [16]. The loss of Tp53 activity in cooperation with EGR1 and Apc haploinsufficiency creates an environment that is permissive for malignant transformation and the development of AML [17]. 2′-Benzoyloxycinnamaldehyde (BCA) induces prostate cancer cell death via EGR1 upregulation and nuclear translocalization, followed by activation of proapoptotic target genes [18].

Increasing evidence suggests that non-coding RNAs have multiple important roles in transcriptional regulation, and also contribute to the expansion of genome complexity. The long noncoding RNA H19 has been implicated in development and growth control and is associated with human genetic disorders and cancer. H19 has been recently characterized as an oncogenic lncRNA in some tumors. An HIV-encoded antisense long noncoding RNA epigenetically regulates viral transcription and overexpression of H19 significantly increased the sphere-forming capacity [19]. H19 RNA expression is to regulate the expression of IGF2 (Insulin Growth Factor 2) [20]. The methylated paternal H19 allele replicates early in the S phase while the hypomethylated maternal allele replicates later, and the later-replicating maternal H19 allele is CTCF-bound [21]. Knock-down of H19 lead to increased polyploidization of mesenchymal stem cells, and induced polyploidy resulted in reduced expression of H19, providing a direct link between H19 expression and the amount of DNA within the cell [22]. H19 is expressed at high levels in cancer cells and increased H19 expression is found in some cancers.e.g. adrenocortical neoplasms, choriocarcinomas, hepatocellular carcinomas, bladder cancers, esophageal cancer and lung cancer [23, 24, 25, 26, 27]. Cells expressing H19 are able to form bigger colonies in soft agar and subcutaneous injection of H19 into mice promoted tumor progression [28]. Downregulation of H19 in breast and lung cancer cells decreases their clonogenicity and anchorage-dependent growth [29]. The effect of H19 in gastric cancer is mediated by the direct upregulation of ISM1 and the indirect suppression of CALN1 expression via miR-675 [30]. In HCC, the expression of H19 and IGF2 usually changes from monoallelic to biallelic. In *in vitro* studies, culturing HCC cell lines in hypoxic condition upregulated H19 expression [31]. In addition, H19 is positively correlated with the presence of steroid receptors, uPar, c-src kinase, tyrosine kinase 2 mitogen-activated protein kinase kinase, tyrosine kinase 2, c-jun, JNK1 [32]. Intriguingly, Maternal imprinting at the H19-Igf2 locus maintains adult haematopoietic stem cell quiescence [33]. The H19 expression is able to function as a cascade activator of trophoblast lineage commitment possibly by overriding the Oct3/4 action in ESCs [34]. H19 modulates let-7 availability by acting as a molecular sponge [35]. Strikingly, H19 depletion results in impaired insulin signaling and decreased glucose uptake [36]. Notably, silencing Mineral dust-induced gene (mdig) increased the level of H3K9me3 in the promoter region of H19 but also attenuated the transcription of H19 long non-coding RNA [37]. Intriguingly, histone H1.3 overexpression leads to increase occupancy of H1.3 at the H19 regulator region encompassing the imprinting control region (ICR) so that H1.3 dramatically inhibits H19 expression, which contributes to the suppression of epithelial ovarian carcinogenesis [38].

Abnormal metabolism and sustained proliferation are hallmarks of cancer. Pyruvate kinase M2 (PKM2) is a metabolic enzyme that plays important roles in both processes. PKM2 is subjected to a complex regulation by both oncogenes and tumour suppressors, which allows for a fine-tone regulation of PKM2 activity. PKM2 possesses protein tyrosine kinase activity and plays a role in modulating gene expression and thereby contributing to tumorigenesis [39]. While dimeric PKM2 diverts glucose metabolism towards anabolism through aerobic glycolysis, tetrameric PKM2 promotes the flux of glucose-derived carbons. Equilibrium of the PKM2 dimers and tetramers is critical for tumorigenesis. PKM2 promotes glucose metabolism and cell growth in gliomas through a mechanism involving a let-7a/c-Myc/hnRNPA1 feedback loop [40]. JMJD5, a Jumonji C domain-containing dioxygenase, interacts directly with pyruvate kinase muscle isozyme (PKM)2 to modulate metabolic flux in cancer cells. The JMJD5-PKM2 interaction resides at the intersubunit interface region of PKM2, which hinders PKM2 tetramerization and blocks pyruvate kinase activity [41]. LPS induces expression of the key metabolic regulator PKM2. PKM2 is therefore a critical determinant of macrophage activation by LPS, promoting the inflammatory response [42]. The binding of PKM2 with TGF-β-induced factor homeobox 2 (TGIF2) recruits histone deacetylase 3 to the E-cadherin promoter sequence, with subsequent deacetylation of histone H3 and suppression of E-cadherin transcription, leading to epithelial-mesenchymal transition [43]. It is long known that PKM2 promotes tumor angiogenesis by increasing endothelial cell proliferation, migration, and cell-ECM adhesion. Only the dimeric PKM2 possess the activity in promoting tumor angiogenesis [44]. The PKM2 knockdown-resistant cells were further subdivided into less glycolytic and more (glycolysis branch pathway-dependent) glycolytic groups [45]. Recently, PKM2 was shown to have protein kinase activity phosphorylating histone H3 and promoting cancer cell proliferation [46]. Regulation of PKM2 activity supports the different metabolic requirements of proliferating and nonproliferating tumor cells [47]. Strikingly, tissue-specific isoform switch and DNA hypomethylation of the pyruvate kinase PKM gene in human cancers [48]. PKM2 is instrumental in both aerobic glycolysis and gene transcription. PKM2 regulates G1-S phase transition by controlling cyclin D1 expression. PKM2 binds to the spindle checkpoint protein Bub3 during mitosis and phosphorylates Bub3 at Y207. Moreover, the level of Bub3 Y207 phosphorylation correlated with histone H3-S10 phosphorylation in human glioblastoma specimens and with glioblastoma prognosis [49].

In this report, we demonstrate miR675 is involved in the epigenetic regulation of H3K9me3, H3K27me3, H3K27Ac for gene expression during hepatocarcinogenesis. miR675 overexpression promotes liver cancer cell growth *in vitro* and *in vivo*. miR675 upregulates lincRNA H19. Strikingly, H19 induces and activates tumor-specific pyruvate kinase M2 (PKM2) which is essential for the warburg effect in its dimer and for gene expression in its teramer during tumorigenesis. These findings sheds light on the significance of miR675-HP1α-EGR1-H19-PKM2 cascade signaling pathway in cancer cells.

## RESULTS

### miR675 promotes liver cancer cells malignant proliferation

**Figure 1:**
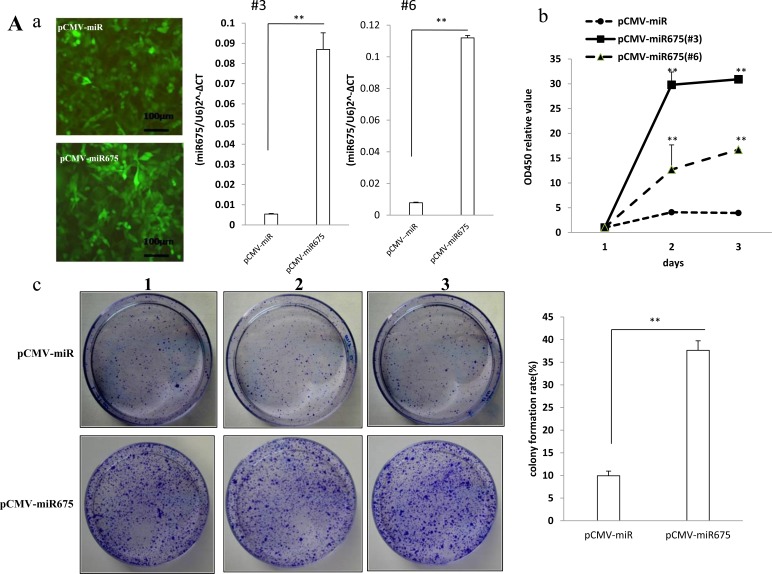

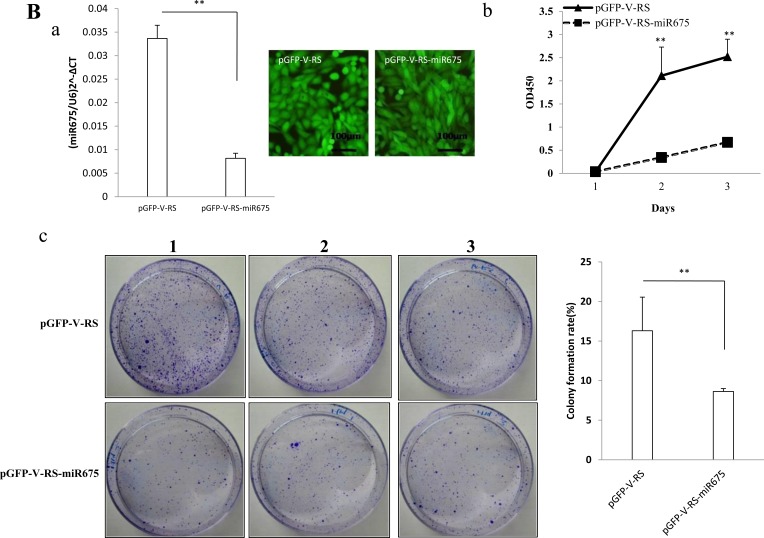

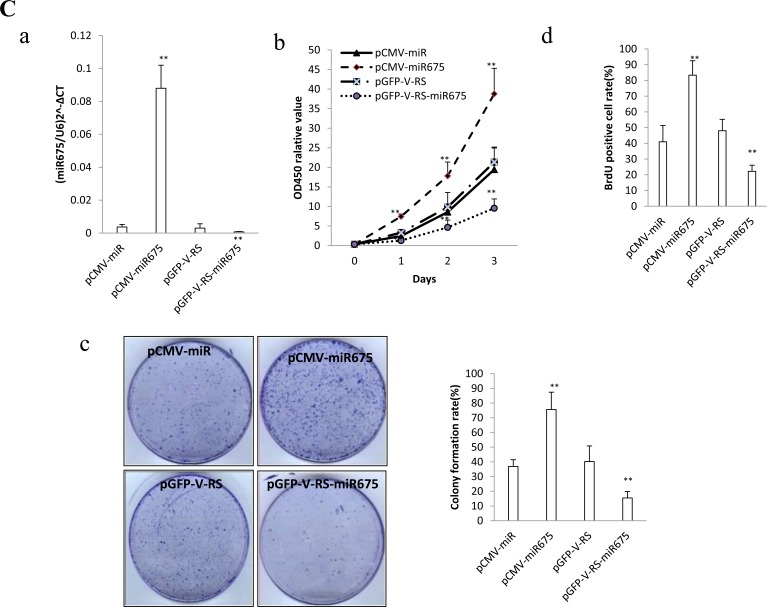
miR675 promotes liver cancer cells proliferation **A.** a. *(left)* The photography of the Hep3B cell lines transfected with pCMV-mir or pCMV-miR675. (*right)*Real-time RT-PCR for mature miR675 in miR675 overexpressed or mock control Hep3B stable cell lines (3# and 6# clone) ;U6 as internal control ;Data are means of value from three independent experiment, bar±SEM. **, *P* < 0.01 ;*, *P* < 0.05. b. Cell proliferation assay was performed in 96-well format using the CCK8 cells proliferation kit to determine the cell viability as described by the manufacturer. Each sample was assayed in triplicates for 3 days consecutively. Cell growth curve was based on the corresponding the relative values of OD450 and each point represents the mean of three independent samples. Data are means of value from three independent experiments, bar±SEM. **, *P* < 0.01 ;*, *P* < 0.05.c. *(right)*Cell plate colony formation ability assay. Data are means of value from three independent experiment, bar±SEM. **, *P* < 0.01 ;*, *P* < 0.05. *(left)*The photography of colonies from the cell lines indicated in *left.* **B.** a. (*Left)*Real-time RT-PCR for mature miR675 in miR675 knocked down or mock control Hep3B stable cell lines ;U6 as internal control; Data are means of value from three independent experiment, bar±SEM. **, *P* < 0.01 *). (right)* The photography of the cell lines transfected with pGFP-V-RS or pGFP-V-RS-miR675.b. Cell proliferation assay was performed in 96-well format using the CCK8 cells proliferation kit to determine the cell viability as described by the manufacturer. Each sample was assayed in triplicates for 3 days consecutively. Cell growth curve was based on the corresponding values of OD450 and each point represents the mean of three independent samples. Data are means of value from three independent experiments, bar±SEM. **, *P* < 0.01 ;*, *P* < 0.05. c. *(right)*Cell plate colony formation ability assay. Data are means of value from three independent experiment, bar±SEM. **, *P* < 0.01 ;*, *P* < 0.05. *(left)*The photography of colonies from the cell lines indicated in *left*. **C.** a. Real-time RT-PCR for mature miR675 in miR675 overexpressed or knocked-down HepG2 stable cell lines;U6 as internal control ;Data are means of value from three independent experiment, bar±SEM. **, *P* < 0.01 ;*, *P* < 0.05. b. Cell proliferation assay was performed in 96-well format using the CCK8 cells proliferation kit to determine the cell viability as described by the manufacturer. Each sample was assayed in triplicates for 3 days consecutively. Cell growth curve was based on the corresponding the relative values of OD450 and each point represents the mean of three independent samples. Data are means of value from three independent experiments, bar±SEM. **, *P* < 0.01 ;*, *P* < 0.05.c. *(left)*Cell plate colony formation ability assay. Data are means of value from three independent experiment, bar±SEM. **, *P* < 0.01 ;*, *P* < 0.05. *(right)*The photography of colonies from the cell lines indicated in *left*. d. Cell BrdU assay. Data are means of value from three independent experiment, bar±SEM. **, *P* < 0.01 ;*, *P* < 0.05.

To address whether the miR675 alters primary liver cancer cells malignant proliferation capacity, we first established the stable human liver cancer cell lines (Hep3B) transfected with pCMV-miR, pCMV-miR675, pGFP-V-RS, pGFP-V-RS-miR675 respectively. We selected the GFP positive cell for screening miR675 overexpression or knockdown stable cell lines (Figure 1A a, left and Figure 1B a, right ). We confirmed mature miR675 expression using real-time RT-PCR and the results showed that mature miR675 was significantly overexpressed in pCMV6-miR675 transfected Hep3B compared with control (*P* < 0.01) and the expression of 3# clone is slight higher compared to 6# (Figure 1A a, right, 3#&6#), while mature miR675 was significantly knocked down in pGFP-V-miR675 transfected Hep3B compared the control (*P* < 0.01) ( (Figure 1Ba, left). At the first time, we detected these cells proliferation capacity *in vitro* using CCK8. As shown in Figure 1Ab, mature miR675 overexpression promoted Hep3B proliferation (the 2^nd^ day & the 3^rd^ day, *P* < 0.01). Strikingly, the growth from 3# clone was significant faster than that from 6# (*P* < 0.01). On the contrast, mature miR675 knockdown inhibited Hep3B proliferation (the 2^nd^ day & the 3^rd^ day, *P* < 0.01) (Figure 1Bb). The colony-formation rate was significantly increased in mature miR675 overexpressed Hep3B compared to control Hep3B (37.63±2.18% *vs* 9.93±1.03%, *P* < 0.01) (Figure 1Ab). In contrast, the plate colony-formation rate was significantly decreased in mature miR675 knocked down Hep3B compared to control Hep3B (16.3±4.26% *vs* 8.63±0.38%, *P* < 0.01) (Figure 1Bc).

Further on, we slected the human liver cancer cell lines HepG2 for *in vitro* tumorigenesis test. Our results showed that mature miR675 was significantly overexpressed in pCMV-miR675 transfected HepG2 compared to control (*P* < 0.01), while mature miR675 was significantly knocked down in pGFP-V-RS-miR675 transfected HepG2 compared to the control (*P* < 0.01) (Figure 1Ca). Mature miR675 overexpression promoted and miR675 knockdown inhibited HepG2 proliferation (*P* < 0.01) (Figure 1Cb), colony formation ability (75.63±11.74% versus 36.93 ±4.5%;15.46±4.35% versus 40.2±10.63%, respectively, *P* < 0.01) (Figure 1Cc), Brdu position rate (83.33± 9.22% versus 41.0±10.35%;22.21±3.90% versus 48.03±7.24%, respectively, *P* < 0.01) (Figure 1Cd). Collectively, these results suggest that miR675 promotes the liver cancer cells malignant proliferation.

### miR675 accelerates liver cancer cells growth *in vivo*

**Figure 2:**
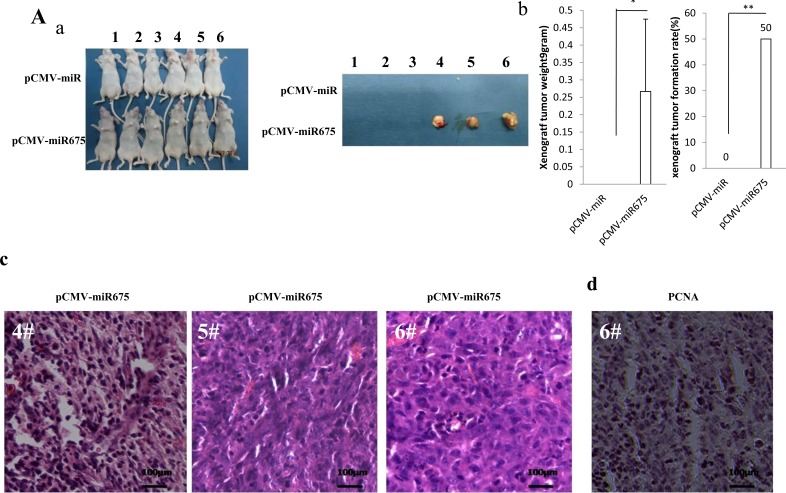

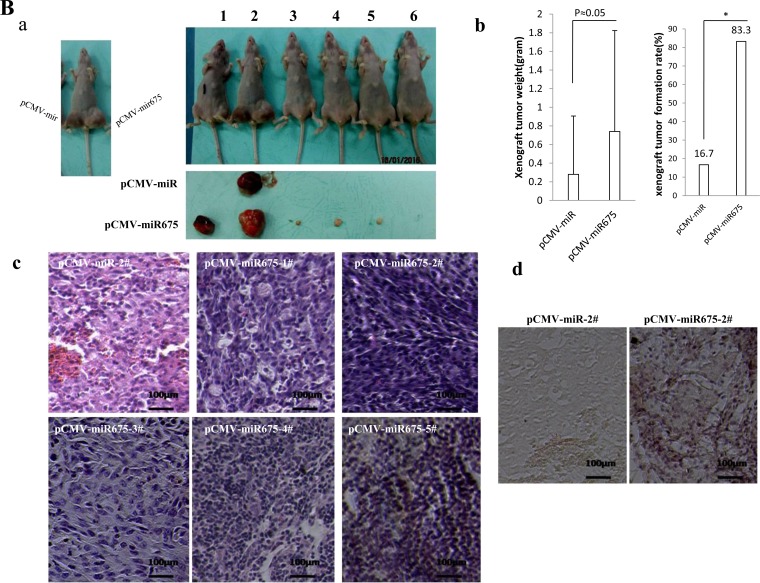

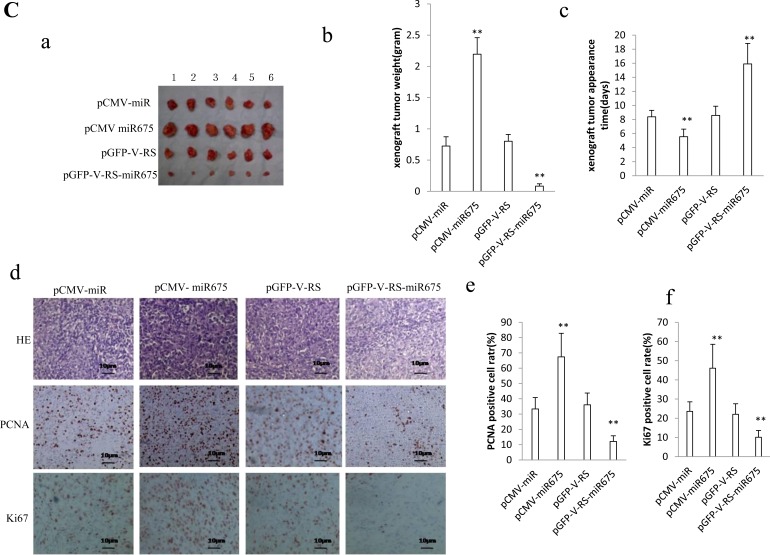
miR675 accelerates liver cancer cell growth *in vivo* **A** a. The photography of xenograft tumors from Balb/C null mouse injected with Hep3B cells transfected with pCMV-miR or pCMV-miR675 subcutaneously at armpit.b. (*left*) The xenograft tumors formation ability (%) in two groups indicated in *lower*. (*right*) The xenograft tumors weight (*gram*) in two groups indicated in *lower*. Data were means of value from six Balb/c mice, mean±SEM, *n* = 6, *, *P* < 0.05;**, *P* < 0.01. c. A portion of each xenograft tumor was fixed in 4% formaldehyde and embedded in paraffin, and the micrometers of sections (4μm) were made for hematoxylin-eosin (HE) staining (original magnification×100). d.anti-PCNA immunostainning in xenograft tumor sample (6#). **B.** a. The photography of xenograft tumors from Balb/C null mouse injected with Hep3B cells transfected with pCMV-miR or pCMV-miR675 subcutaneously at armpit. b. The xenograft tumors weight (*gram*) in two groups indicated in *left*. Data were means of value from six Balb/c mice, mean±SEM, *n* = 6, *, *P* < 0.05;**, *P* < 0.01. c. A portion of each xenograft tumor was fixed in 4% formaldehyde and embedded in paraffin, and the micrometers of sections (4μm) were made for hematoxylin-eosin (HE) staining (original magnification×100). *d.* anti-PCNA immunostainning in xenograft tumor sample (pCMV-miR *2#* and pCMV-miR675 *2#).* **C.** a. The photography of xenograft tumors from Balb/C null mouse injected with HepG2 cells transfected with pCMV-miR, pCMV-miR675, pGFP-V-RS or pGFP-V-RS-miR675subcutaneously at armpit. b. The xenograft tumors weight (*gram*) in the four groups indicated in *left*. Data were means of value from six Balb/c mice, mean±SEM, *n* = 6, *, *P* < 0.05;**, *P* < 0.01. c. A portion of each xenograft tumor was fixed in 4% formaldehyde and embedded in paraffin, and the micrometers of sections (4μm) were made for hematoxylin-eosin (HE) staining (original magnification×100). *d.* anti-PCNA and anti-k67 immunostainning in xenograft tumor samples.

Given that the miR675 promotes liver cancer cells malignant proliferation, we further consider to identity the effect of miR675 on hepatocarcinogenesis *in vivo*. The Hep3B stable cell lines with altered expression of mature miR675 were injected subcutaneously into Balb/C nude mice. There were 36 cases of Balb/C mice in first animal tumorigenesis test in *vivo*. As shown in Figure 2A (a, b), when mature miR675 was overexpressed, the xenograft tumor were produced in three mice (0.1gram, 0.2gram, 0.5gram, respectively), while there was no xenograft tumor in control group (0.2667±0.2081 gram *vs* 0 gram, *n* = 3, *P* = 0.045376) (Figure 2Ab, left). The tumor formation ability in miR675 overexpressed group is significant higher than in control group (50% *vs* 0%, *n* = 6, *P* = 0.00617) (Figure 2Ab, right), On the other hand, xenograft tumor was not produced in either mature miR675 knocked-down or RNAi control group. Pathological picture (hematoxylin-eosin staining) of three xenograft tumors from mature miR675 overexpressed group showed these tissue possessed poor-differentiation cells (5# & 6# xenograft tumors) or less moderately cancer cells (4# xenograft tumors) (Figure 2Ac), and stronger Proliferating cell nuclear antigen (PCNA) positive staining in 6# xenograft (Figure 2Ad). There were 6 cases of Balb/C nude mice in second animal test in *vivo*. for each Balb/C mouse, miR675 overexpressed Hep3B cells were injected into *right lower* armpit area, miR control Hep3B cells were injected into *left lower* armpit area, miR675 knocked-down Hep3B cells were injected into *right upper* armpit area, RNAi control Hep3B cells were injected into *left upper* armpit area. As shown in Figure 2B (a, b), when mature miR675 was overexpressed, the xenograft tumors were produced in five mice (2.6gram, 0.8gram, 0.1gram, 0.1gram, 0.1gram respectively), while there was one xenograft tumor in control group (1.4gram) (0.28±0.62 gram *vs* 0.74±1.08 gram, *n* = 5, *P* ≈ 0.05) (Figure 2Bb, left). The tumor formation ability in miR675 overexpressed group is significant higher than in control group (83.3% *vs* 16.7%, *n* = 6, *P* = 0.0374) Figure 2Bb, right). On the other hand, xenograft tumor was not produced in either mature miR675 knocked-down or control group. Pathological picture (hematoxylin-eosin staining) of xenograft tumors from mature miR675 overexpressed group showed these tissue possessed poor-differentiation cells (1#, 2#, 4# xenograft tumors) or less moderately cancer cells (3# & 5# xenograft tumors), and pathological picture of xenograft tumor from control group showed these tissue possessed well-differentiation cells (control 1# xenograft tumor) (Figure 2Bc), and stronger Proliferating cell nuclear antigen (PCNA) positive staining in 2# miR675 xenograft tumor compared to 2# control xenograft tumor (Figure 2Bd).

As for HepG2 cell lines, there were 24 Balb/C nude mice in the third animal test in *vivo*. As shown in Figure 2Ca, when mature miR675 was overexpressed, the xenograft tumor weight increased approximately three folds when compared to the corresponding control group (2.195±0.265 grams versus 0.725±0.148 grams, *P* = 0.000018). On the other hand, when mir675 was knocked down, the average xenograft tumor weight decreased to approximately one tenth of the control weight (0.083±0.036 grams versus 0.802±0.108 grams, *P* = 0.0000047) (Figure 2Cb). Mature miR675 overexpression resulted in early xenograft tumor formation compared to the control group (5.55±1.08 days versus 8.38±0.92 days, *P* = 0.0050966). In contrast, the time of xenograft tumor appearance was prolonged in the mir675 knockdown group compared to the control group (15.91±2.92 days versus 8.58±1.31 days, *P* = 0.001512) (Figure 2Cc). The xenograft tumor showed that tumor tissue possessed more poor-differentiation cells in mature mir675 overexpression group than that of control group, and less poor-differentiation cells in mir675 knockdown group than that of control group (Figure 2Cd upper). The PCNA-positive cells was significantly higher in miR675 overexpressed tumors compared to the vector control (67.42±15.37% versus 33.33±7.47%, *P* = 0.0023). Conversely, the percentage of PCNA positive cells was significantly lower in miR675 knockdown tumors (12.08±3.74% versus 36.05±7.69%, *P* = 0.000704) (Figure 2Cd middle and 2Ce). The Ki67-positive cells was significantly higher in miR675 overexpressed tumors compared to the vector control (46.07±12.4% versus 23.58±4.99%, *P* = 0.003784). Conversely, the percentage of Ki67 positive cells was significantly lower in miR675 knockdown tumors (10.11±3.51% versus 22.56±5.49%, *P* = 0.000959) (Figure 2Cd lower and 2Cf). Taken together, these observations demonstrate that miR675 accelerates liver cancer growth *in vivo*.

### miR675 inhibits HP1 isoforms (HP1α, HP1β, HP1γ) expression

**Figure 3:**
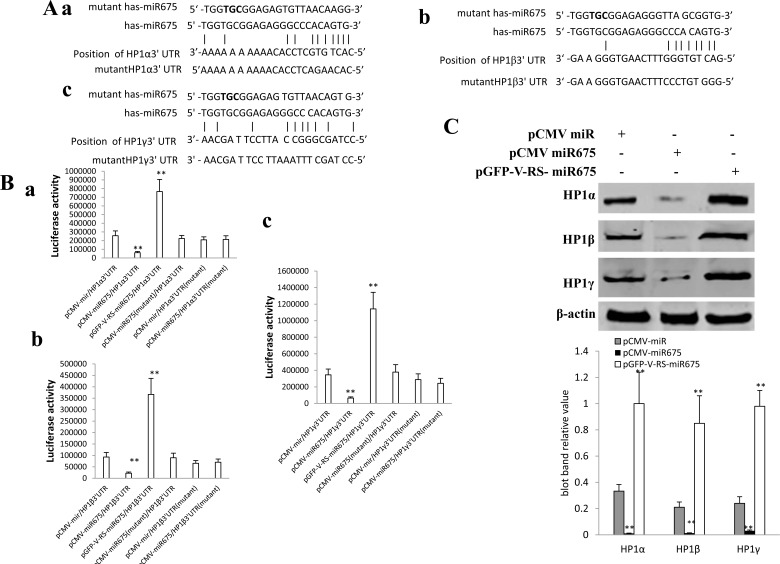

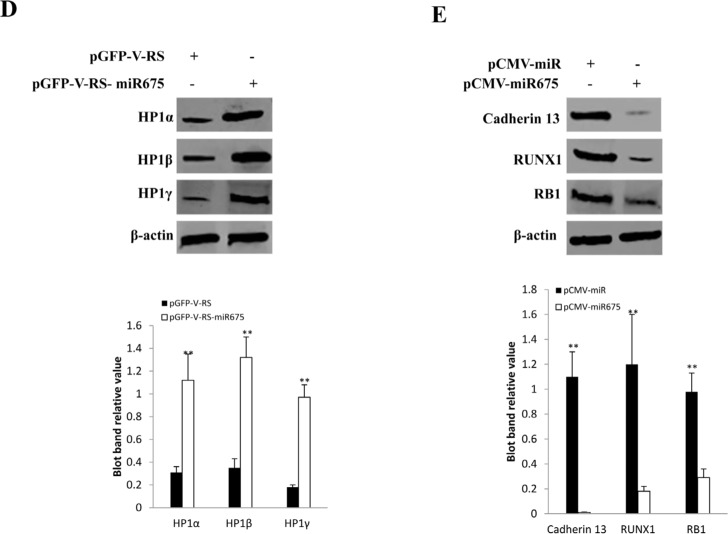
miR675 inhibits HP1 α, HP1 β, HP1 γ expression in human liver cancer cells **A.** MirTarget bioinformatics analysis. a. Mir675 targets for human HP1 α 3′UTR ; b. Mir675 targets for huamn HP1 β 3′UTR;c. Mir675 targets for HP1 γ 3′UTR. **B.** HP1α, β, γ-3′-UTR-Luciferase activity assay. Hep3B cells were transfected with pMirTarget-control, pMirTarget-HP1α3′UTR, pMirTarget-mutant HP1α3′UTR, pMirTarget-HP1β3′UTR, pMirTarget-mutant HP1β3′UTR, pMirTarget-HP1γ3′UTR, pMirTarget-mutant HP1γ3′UTR, pCMV-miR, pCMV-miR675, pCMV-mutant miR675 respectively. Data are means of value from three independent experiment, bar±SEM. **, *P* < 0.01. **C.** Western blotting analysis using anti-HP1 α, anti-HP1β, anti-HP1γ in Hep3B cell lines transfected with pCMV-miR, pCMV-miR675, pGFP-V-RS-miR675, respectively.β-actin as internal control. **D.** Western blotting analysis using anti-HP1 α, anti-HP1β, anti-HP1γ in Hep3B cell lines transfected with pGFP-V-RS, pGFP-V-RS-miR675, respectively.β-actin as internal control. C. Western blotting analysis using anti-HP1 α, anti-HP1β, anti-HP1γ in Hep3B cell lines transfected with pCMV-miR, pCMV-miR675, pGFP-V-RS-miR675, respectively.β-actin as internal control. D. Western blotting analysis using anti-Cadherin 13, anti-RUNX1, anti-RB1 in Hep3B cell lines transfected with pCMV-miR, pCMV-miR675, respectively.β-actin as internal control.

To address whether miR675 targets for HP1α, β, γ and influences on their expression, we first performs the informatics analysis using MirTarget scanning soft (predicts biological targets of miRNAs by searching for the presence of conserved 8mer and 7mer sites that match the seed region of each miRNA) and BLAST analysis. As shown in Figure 3A, mature miR675 can match 3′ untranslational region on HP1α mRNA (BC006821) via eleven seed sequence (a); mature miR675 can match 3′ untranslational region on HP1β mRNA (BC002609) via nine seed sequence (b);mature miR675 can match 3′ untranslational region on HP1γ mRNA (AB030905) via ten seed sequence (c). Next, we constructed the luciferase report vector consisting of HP1α/β/γ 3′UTR containing miR675 targeting site and their corresponding mutant plasmid of HP1α/β/γ 3′UTR, and pCMV-miR mutant miR675. As shown in Figure 3B, the HP1α3′UTR luciferase activity was significantly reduced in mature miR675 overexpressed Hep3B cells compared to control group (*p* < 0.01) and increased in mature miR675 knocked down Hep3B cells compared to control group (*p* < 0.01). Nevertheless, the HP1α3′UTR luciferase activity was significantly not altered in Hep3B cell lines transfected with pCMV-mutant miR675 plus pMirtarget-HP1α3′UTR, pMirtarget-mutant HP1α3′UTR or pCMV-miR675 plus pMirtarget-mutant HP1α3′UTR (*P* > 0.05). The HP1β 3′UTR luciferase activity was significantly reduced in mature miR675 overexpressed Hep3B cells compared to control group (*p* < 0.01) and increased in mature miR675 knocked down Hep3B cells compared to control group (*p* < 0.01). Nevertheless, the HP1β3′UTR luciferase activity was significantly not altered in Hep3B cell lines transfected with pCMV- mutant miR675 plus pMirtarget-HP1β3′UTR, pMirtarget-mutant HP1β3′UTR or pCMV- miR675 plus pMirtarget-mutant HP1β3′UTR (*P* > 0.05). The HP1γ3′UTR luciferase activity was significantly reduced in mature miR675 overexpressed Hep3B cells compared to control group (*p* < 0.01) and increased in mature miR675 knocked down Hep3B cells compared to control group (*p* < 0.01). Nevertheless, the HP1γ3′UTR luciferase activity was significantly not altered in Hep3B cell lines transfected with pCMV- mutant miR675 plus pMirtarget-HP1γ3′UTR, pMirtarget-mutant HP1γ3′UTR or pCMV- miR675 plus pMirtarget-mutant HP1γ3′UTR (*P* > 0.05). Then we performed the western blotting using anti-HP1α, anti-HP1β and anti-HP1γ. As shown in Figure 3C, HP1α, HP1β, HP1γ expression were significantly decreased in mature miR675 overexpressed Hep3B cells compared to control group (pCMV-miR). HP1α, HP1β, HP1γ expression were significantly increased in miR675 knockdown Hep3B cells compared to control group (pGFP-V-RS) (Figure 3D). Moreover, our findings showed that mature miR675 overexpression reduced the expression of Cadherin 13, RUNX1 and RB1 in Hep3B cell lines (Figure 3E). Collectively, our findings suggest miR675 targets for HP1 isoforms including HP1α, HP1β, HP1γ and inhibits their expression in liver cancer cells.

### miR675 induces EGR1 expression through reducing HP1α

**Figure 4:**
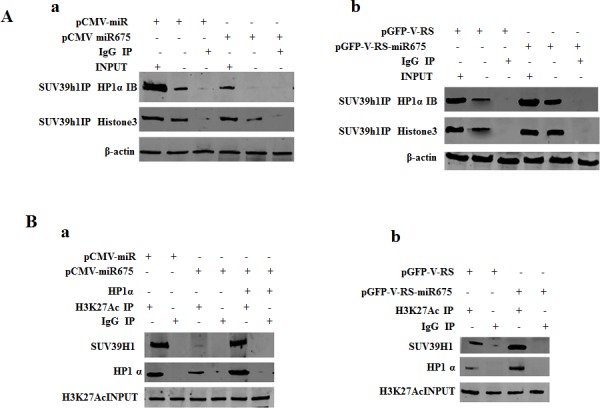

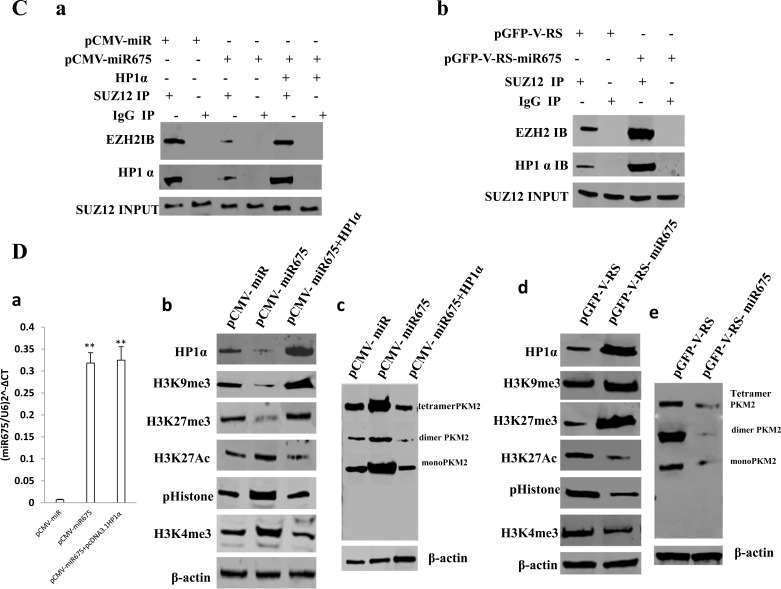

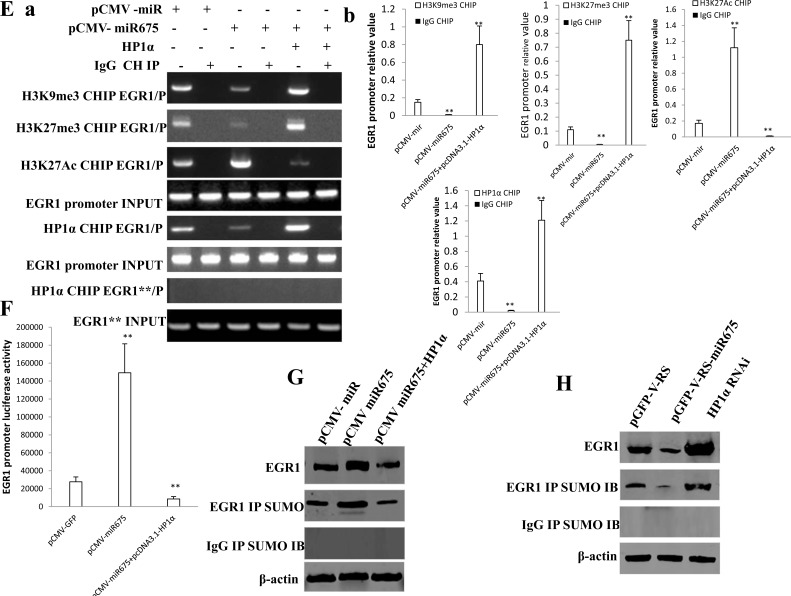
miR675 enhances EGR1 via reducing HP1 α in human liver cancer cells **A.** a. Co-Immunoprecipitation (IP) with anti-SUV39h1 followed by western blotting with anti-HP1α or anti-Histone3 in miR675 overexpressied and control Hep3B. IgG IP as negative control. INPUT refers to western blotting with anti-HP1α. β-actin as internal control. b. Co-Immunoprecipitation (IP) with anti-SUV39h1 followed by western blotting with anti-HP1α or anti-Histone3 in miR675 knockdown and control Hep3B. IgG IP as negative control. INPUT refers to western blotting with anti-HP1α. β-actin as internal control. **B.** a. Co-Immunoprecipitation (IP) wih anti-H3k27Ac followed by western blotting with anti-SUV39H1 in miR675 overexpressied, miR675 plus HP1α overexpressed and control Hep3B. IgG IP as negative control. INPUT refers to western blotting with anti-SUV39h1. b. Co-Immunoprecipitation (IP) wih anti-H3k27Ac followed by western blotting with anti-SUV39H1 in miR675 knockdown control Hep3B. IgG IP as negative control. INPUT refers to western blotting with anti-SUV39h1. **C.** a. Co-Immunoprecipitation (IP) with anti-SUZ12 followed by western blotting with anti-EZH2, anti-HP1α in miR675 overexpressied, miR675 plus HP1α overexpressed and control Hep3B. IgG IP as negative control. INPUT refers to western blotting with anti-EZH2. b. Co-Immunoprecipitation (IP) wih anti-SUZ12 followed by western blotting with anti-EZH2, anti-HP1α in miR675 knockdown and control Hep3B. IgG IP as negative control. INPUT refers to western blotting with anti-EZH2. **D.** a. Real-time RT-PCR for mature miR675 in Hep3B cells transfected with pCMV-miR, pCMV-miR675, pCMV-miR675 plus pcDNA3.1-HP1α respectively. U6 as internal control. b. Western blotting with anti-HP1α, anti-H3K9me3, anti-H3K27me3, anti-pHistone3, H3K27Ac, anti-H3K4me3 in Hep3B cells transfected with pCMV-miR, pCMV-miR675, pCMV-miR675 plus pcDNA3-HP1α respectively. β-actin as internal control. c. Western blotting with anti-PKM2 in Hep3B cells transfected with pCMV-miR, pCMV-miR675, pCMV-miR675 plus pcDNA3-HP1α respectively. β-actin as internal control. d. Western blotting with anti-HP1α, anti-H3K9me3, anti-H3K27me3, anti-pHistone3, H3K27Ac, anti-H3K4me3 in Hep3B cells transfected with pGFP-V-RS, pGFP-V-RS-miR675 respectively. β-actin as internal control. e. Western blotting with anti-PKM2 in Hep3B cells transfected with pGFP-V-RS, pGFP-V-RS-miR675 respectively. β-actin as internal control. **E.** a. Chromatin Immunoprecipitation (CHIP) with anti-H3K9me3, anti-H3K27me3, anti-H3K27Ac and anti-HP1α followed by PCR with EGR1 promoter primers in miR675 overexpressied, miR675 pluc HP1α overexpressed and control Hep3B. IgG IP as negative control. IgG CHIP as negative control. EGR1 promoter DNA sequence as INPUT. EGR1 DNA** as negative control.b. Chromatin Immunoprecipitation (CHIP) with anti-H3K9me3, anti-H3K27me3, anti-H3K27Ac and anti-HP1α followed by real-time PCR with EGR1 promoter primers in miR675 overexpressied, miR675 pluc HP1α overexpressed and control Hep3B. **F.** EGR1 promoter luciferase activity assay in Hep3B cells transfected with pCMV-miR, pCMV-miR675, pCMV-miR675 plus pcDNA3-HP1α respectively. Each value was presented as mean±standard error of the mean (SEM). **G.** Western blotting with anti-EGR1 and CO-IP with anti-SUM (EGR1 IB) in Hep3B cells transfected with pCMV-miR, pCMV-miR675, pCMV-miR675 plus pcDNA3-HP1α respectively. β-actin as internal control. **H.** Western blotting with anti-EGR1 and CO-IP with anti-SUM (EGR1 IB) in Hep3B cells transfected with pGFP-V-RS, pGFP-V-RS-miR675, pGFP-V-RS- HP1α respectively. β-actin as internal control.

We infer miR675 may alter early growth response protein1 (EGR1) expersion epigenetically. To explore whether mature miR675 alters EGR1 expression, we consider to reveal whether mature miR675 impacts on the Histone 3 modification in liver cancer cells. At the first time, we performed the Co-immunoprecipitation (Co-IP) to analyse the interaction between SUV39h1 and HP1α, SUV39h1 and Histone3, SUV39H1 and H3K27Ac, EZH2 and SUZ12 in Hep3B cells transfected with pCMV-miR, pCMV-miR675, pCMV-miR675 plus pcDNA3.1-HP1α. Our results showed that the interplay between SUV39H1 and HP1α, SUV39H1 and Histone3 was attenuated in mature miR675 overexpressed Hep3B compared to control group (Figure 4Aa) and that the interplay between SUV39H1 and HP1α, SUV39H1 and Histone3 was increased in mature miR675 knockdown Hep3B compared to control group (Figure 4Ab). Obviously, this is due to a reduction of HP1 expression in miR675 overexpressed Hep3B. As shown in Figure 4Ba, the interplay between SUV39H1 and H3K27Ac, H3K27Ac and HP1α were weakened in mature miR675 overexpressed Hep3B compared to control group. However, the decreased interaction between SUV39H1 and H3K27Ac was rescued and interaction between H3K27Ac and HP1α was slightly increased in Hep3B cells transfected with pCMV-miR675 plus pcDNA3.1-HP1α compared to the control. The interplay between SUV39H1 and H3K27Ac, H3K27Ac and HP1α were enhanced in mature miR675 knockdown Hep3B compared to control group (Figure 4Bb). In addition, the interplay between EZH2 and SUZ12, SUZ12 and HP1α were attenuated in mature miR675 overexpressed Hep3B compared to control group. However, this interaction between EZH2 and SUZ12 was not altered and this interaction between SUZ12 and HP1α was increased in Hep3B cells transfected with pCMV-miR675 plus pcDNA3.1-HP1α compared to the control (Figure 4Ca). The interplay between EZH2 and SUZ12, SUZ12 and HP1α were increased in mature miR675 knockdown Hep3B compared to control group (Figure 4Cb). Further on, we detected the Histone3 modification (H3K9me3, H3K27me3, H3K27Ac, pHistone3 and H3K4me3) and PKM2 expression in liver cancer. In Hep3B cells, mature miR675 was significantly overexpressed in Hep3B cell lines transfected with pCMV-miR675 or pCMV-miR675 plus pcDNA3.1-HP1α compared to control (Figure 4Da). HP1α expression was significantly reduced in Hep3B cell line transfected with pCMV-miR675 and significantly overexpressed in Hep3B cell line transfected with pCMV-miR675 plus pcDNA3.1-HP1α compared to control (Figure 4Db, the first row from upper to lower). Both H3K9me3 and H3K27me3 were significantly decreased in Hep3B cell line transfected with pCMV-miR675. Notably, both H3K9me3 and H3K27me3 were slightly increased in Hep3B cell line transfected with pCMV-miR675 plus pcDNA3.1-HP1α compared to control (Figure 4Db, the second & third row from upper to lower). H3K27Ac, pHistone3 and H3K4me3 were significantly increased in Hep3B cell line transfected with pCMV-miR675. It was worth noting that H3K27Ac and H3k4me3 was slightly decreased and pHistone was not altered in Hep3B cell line transfected with pCMV-miR675 plus pcDNA3.1-HP1α compared to control (Figure 4Db, the forth, fifth, sixth row from upper to lower). Strikingly, mono PKM2 (58KD), PKM2 dimer (116KD) and PKM2 tertamer (232KD) were significantly increased in Hep3B cell line transfected with pCMV-miR675. However, mono PKM2 (58KD), PKM2 dimer (116KD) and PKM2 tertamer (232KD) were significantly reduced in Hep3B cell line transfected with pCMV-miR675 plus pcDNA3.1-HP1α compared to control (Figure 4Dc). HP1α expression was significantly increased in Hep3B cell line transfected with pGFP-V-RS-miR675 compared to control (Figure 4Dd, the first row from upper to lower). Both H3K9me3 and H3K27me3 were significantly increased in Hep3B cell line transfected with pGFP-V-RS-miR675 compared to control (Figure 4Dd, the second & third row from upper to lower). H3K27Ac, pHistone3 and H3K4me3 were significantly decreased in Hep3B cell line transfected with pGFP-V-RS-miR675 compared to control (Figure 4Dd, the forth, fifth, sixth row from upper to lower). Strikingly, mono PKM2 (58KD), PKM2 dimer (116KD) and PKM2 tertamer (232KD) were significantly decreased in Hep3B cell line transfected with pGFP-V-RS-miR675 compared to control (Figure 4De). Next, we performed the Chromatin Immunoprecipitation (CHIP) assay in liver cancer cell line. As shown in Figure 4E (a, b), the loading of H3Kme9, H3K27me3, HP1α on the EGR1 promoter region were attenuated in Hep3B cell line transfected with pCMV-miR675. However, the loading of H3Kme9, H3K27me3, HP1α on the EGR1 promoter region were enhanced in Hep3B cell line transfected with pCMV-miR675 plus pcDNA3.1-HP1α compared to control. By contraries, the loading of H3K27Ac on the EGR1 promoter region were increased in Hep3B cell line transfected with pCMV-miR675 compared to control. It is noteworthy that the loading of H3K27Ac on the EGR1 promoter region were slightly weakened in Hep3B cell line transfected with pCMV-miR675 plus pcDNA3.1-HP1α compared to contro1. Then we performed the luciferase activity assay in aforementioned Hep3B.cell lines. As shown in Figure 4F, EGR1 promoter luciferase activity was significantly increased in Hep3B cell line transfected with pCMV-miR675 compared to control (149207.3±32350.21 *vs* 27669.1±5418.8, *P* < 0.01). However, EGR1 promoter luciferase activity was slightly decreased in Hep3B cell line transfected with pCMV-miR675 plus pcDNA3.1-HP1α compared to control (8549.0±2267.5 *vs* 27669.1±5418.8, *P* < 0.01). Finally, we analyse the EGR1 expression by western blotting and EGR1 sumoylation by Co-IP in above-mentioned Hep3B cell lines. As showed in Figure 4G, both EGR1 expression and SUM-EGR1 were significantly increased in Hep3B cell line transfected with pCMV-miR675 compared to control. However both EGR1 expression and SUM-EGR1 were slightly decreased in Hep3B cell line transfected with pCMV-miR675 plus pcDNA3.1-HP1α compared to control. Significantly, both EGR1 expression and SUM-EGR1 were significantly decreased in Hep3B cell line transfected with pGFP-V-RS-miR675 compared to control. Moreover, both EGR1 expression and SUM-EGR1 were significantly increased in Hep3B cell line transfected with pGFP-V-RS-HP1α compared to control (Figure 4H). Taken together, these observations suggest that mature miR675 reduced the complex output of HP1α-SUV39H1-Histone3, HP1α-SUV39H1-H3K27Ac, HP1α-SUZ12-EZH2 which caused the reduction of H3K9me3, H3K27me3 and increment of H3K27Ac, and increased EGR1 turnout through transcriptional regulation ultimately.

### miR675 upregulates H19 expression by increasing the EGR1 occupancy on H19 imprinting control region (ICR)

**Figure 5:**
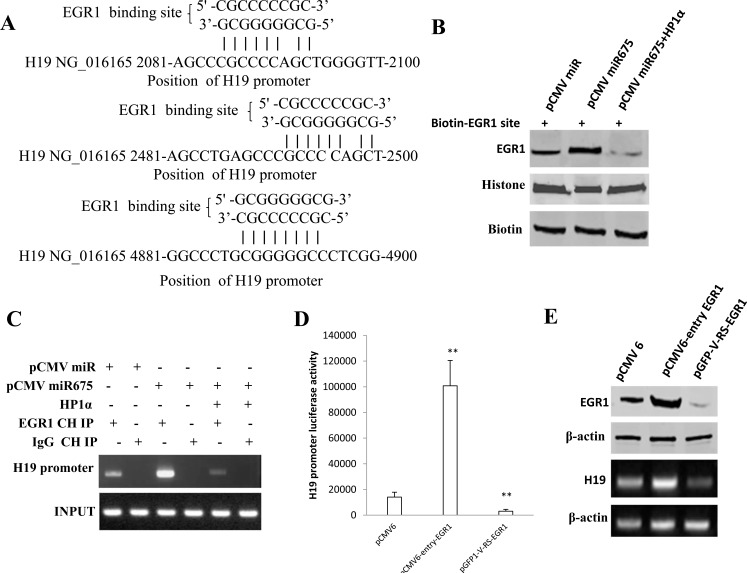

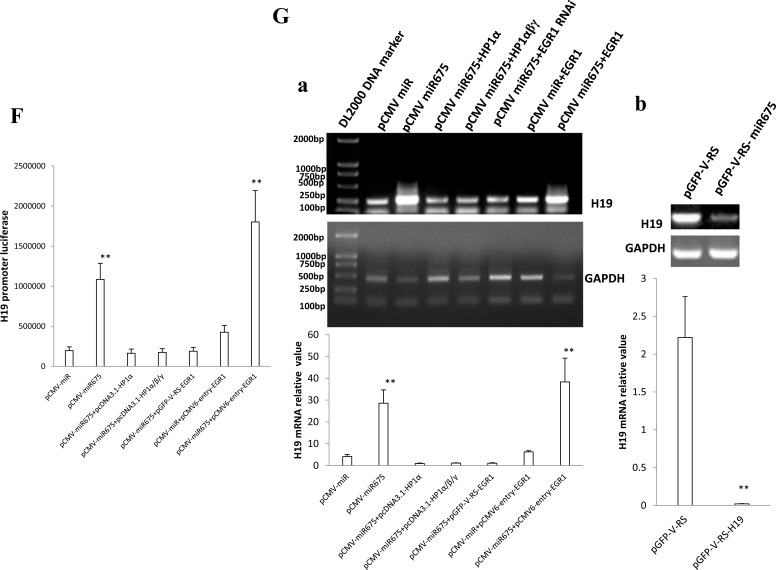
miR675 upregulates H19 through EGR1 activation **A.** informatices analysis:EGR1 site (5′-CGCCCCCGC-3′) and 5′-GCGGGGGCG-3′ on H19 promoter region. **B.** DNA pulldown with Biotin-EGR1 followed by Western blotting with anti-EGR1in Hep3B transfected with pCMV-miR, pCMV-miR675, pCMV-miR675 plus pcDNA3.1-HP1α. Histone as internal control. Biotin as INPUT. **C.** Chromatin Immunoprecipitation (CHIP) with anti-EGR1 followed by PCR with H19 promoter primers in Hep3B transfected with pCMV-miR, pCMV-miR675, pCMV-miR675 plus pcDNA3.1-HP1α. IgG IP as negative control. H19 promoter DNA sequence as INPUT. **D.** H19 promoter luciferase activity assay in Hep3B cells transfected with pCMV6-entery, pCMV6-entry-EGR1, pGFP-V-RS-EGR1 respectively. Each value was presented as mean±standard error of the mean (SEM). **E.** Western blotting for EGR1 and RT-PCR analysis for H19 in Hep3B transfected with pCMV6-AC-GFP, pCMV6-AC-GFP-EGR1, pGFP-V-RS-EGR1 respectively. β-actin as internal control. **F.** H19 promoter luciferase activity assay in Hep3B transfected with pCMV-miR, pCMV-miR675, pCMV-miR675 plus pcDNA3.1HP1α, pCMV-miR675 plus pcDNA3.1HP1αβγ, pCMV-miR675 plus pGFP-V-RS- EGR1, pCMV-miR plus pCMV6-entry-EGR1, pCMV-miR675 plus pCMV6-entry-EGR1 respectively. Each value was presented as mean±standard error of the mean (SEM). **G.** a. RT-PCR analysis for H19 in Hep3B transfected with pCMV-miR, pCMV-miR675, pCMV-miR675 plus pcDNA3.1HP1α, pCMV-miR675 plus pcDNA3.1HP1αβγ, pCMV-miR675 plus pGFP-V-RS- EGR1, pCMV-miR plus pCMV6-entry-EGR1, pCMV-miR675 plus pCMV6-entry-EGR1 respectively. β-actin as internal control. b. RT-PCR analysis for H19 in Hep3B transfected with pGFP-V-RS, pGFP-V-RS--miR675 respectively. β-actin as internal control.

To explore whether miR675 alters long noncoding RNA H19 expression through EGR1 action, we have a reason to consider whether EGR1 may control H19 transcriptional activity. Bioimformatics analysis suggests that EGR1 binding site (5′-CGCCCCCGC-3′ and 5′-GCGGGGGCG-3′ ) exists on the H19 promoter region (Figure 5A). Next, we performed the DNA pulldown assay using the EGR1 site probe. As shown in Figure 5B, The binding capacity of EGR1 to EGR1 site was significantly enhanced in Hep3B cell line transfected with pCMV-miR675 compared to control. However, The binding capacity of EGR1 to EGR1 site was slightly decreased in Hep3B cell line transfected with pCMV-miR675 plus pcDNA3.1-HP1α compared to control to control. Next, we performed the Chromatin Immunoprecipitation (CHIP) assay in Hep3B liver cancer cell line. As shown in Figure 5C, the loading of EGR1 on the H19 promoter region was increased in Hep3B cell line transfected with pCMV-miR675. However, the loading of EGR1 on the H19 promoter region was slightly attenuated in Hep3B cell line transfected with pCMV-miR675 plus pcDNA3.1-HP1α compared to control. In addition, as shown in Figure 5D, H19 promoter luciferase activity was significantly increased in Hep3B cell line transfected with pCMV-entry-EGR1 compared to control (100779.1±19715.4 *vs* 14039.0±3835.9, *P* < 0.01). However, H19 promoter luciferase activity was significantly decreased in Hep3B cell line transfected with pGFP-V-RS-EGR1 compared to control (3058.7±1442.5 *vs* 14039.0±3835.9, *P* < 0.01). Further on, as shown in Figure 5E, both EGR1 expression. and H19 expression were significantly increased in Hep3B cell line transfected with pCMV-entry-EGR1 compared to control. In contrary, both EGR1 expression. and H19 expression were significantly decreased in Hep3B cell line transfected with pGFP-V-RS-EGR1 compared to control. It suggests EGR1 can enhance the H19 transcriptional activity. Strikingly, H19 promoter luciferase activity was significantly increased in Hep3B cells transfected with pCMV-miR675 (1084247.1±201941.8 *vs* 195688.2±47675.9, *P* < 0.01), pCMV-miR675 plus pCMV6-entry-EGR1 (1802573.2±389872.4 *vs* 195688.2±47675.9, *P* < 0.01 ) and pCMV-miR plus EGR1 (426217.7±85.85.2 *vs* 195688.2±47675.9, *P* < 0.01) compared to control. However, H19 promoter luciferase activity was significantly not altered in Hep3B cells transfected with pCMV-miR675 plus pcDNA3.1-HP1α (163712.2±51604.6 *vs* 195688.2±47675.9, *P* > 0.05), pCMV-miR675 plus pcDNA3.1-HP1α, β, γ (174387.3±46190.9 *vs* 195688.2±47675.9, *P* > 0.05) and pCMV-miR675 plus pGFP-V-RS-EGR1 (189216.3±47906.6 *vs* 195688.2±47675.9, *P* > 0.05) compared to control (Figure 5F). Finally, we performed the RT-PCR in liver cancer line. As shown in Figure 5Ga, the H19 mRNA expression was significantly increased in Hep3B cells transfected with pCMV-miR675 and pCMV-miR675 plus pCMV6-entry-EGR1 compared to control. However, H19 mRNA was significantly not altered in Hep3B cells transfected with pCMV-miR675 plus pcDNA3.1-HP1α, pCMV-miR675 plus pcDNA3.1-HP1α, β, γ and pCMV-miR675 plus pGFP-V-RS-EGR1 compared to control. Moreover, the H19 mRNA expression was significantly drcreased in Hep3B cells transfected with pGFP-V-RS-miR675 compared to pGFP-V-RS control (Figure 5Gb). Collectively, these observations strongly suggest that miR675 promotes H19 expression through upregulating and activating EGR1 by targeting for HP1 isoforms.

### miR675 accelerates hepatocarcinogenesis via miR675-HP1α-EGR1-H19-PKM2 axis

**Figure 6:**
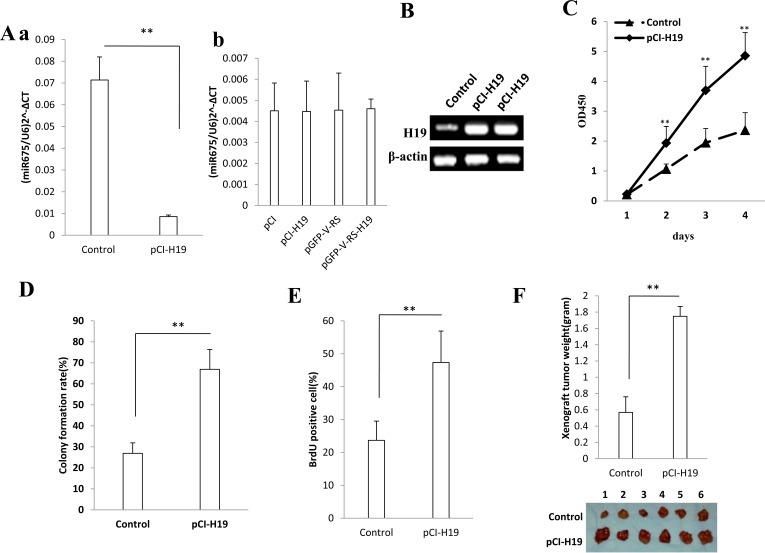

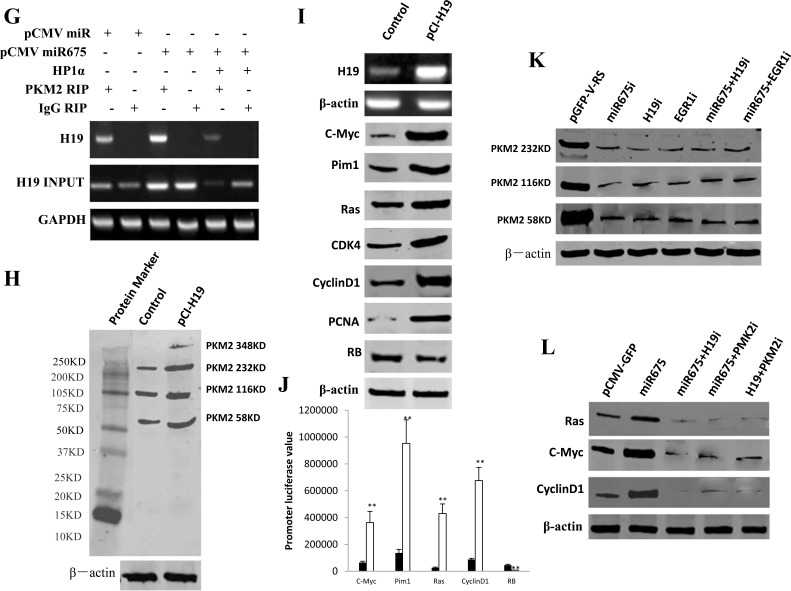
H19 upregulates and activates PKM2 to promote hepatocarcinogenesis **A.** a. real-time RT-PCR analysis for mature miR675 in Hep3B transfected with pCI-control, pCI-H19. U6 as internal control. b. real-time RT-PCR analysis for mature miR675 in HepG2 transfected with pCI-control, pCI-H19, pGFP-V-RS, pGFP-V-RS-H19. U6 as internal control. **B.** RT-PCR analysis for H19 in Hep3B transfected with pCI-H19. β-actin as internal control. **C.** Cell proliferation assay was performed in 96-well format using the CCK8 cells proliferation kit to determine the cell viability as described by the manufacturer. Each sample was assayed in triplicates for 3 days consecutively. Cell growth curve was based on the corresponding the relative values of OD450 and each point represents the mean of three independent samples. Data are means of value from three independent experiments, bar±SEM. **, *P* < 0.01 ;*, *P* < 0.05. **D.** Cell plate colony formation ability assay. Data are means of value from three independent experiment, bar±SEM. **, *P* < 0.01 ;*, *P* < 0.05. **E.** S phase cells assay using BrdU. Each value was presented as mean±standard error of the mean (SEM). **F.** Tumorigenesis test *in vivo*. Xenograft weight are means of value from six Balb/C nude mice, bar±SEM. **, *P* < 0.01 ;*, *P* < 0.05. **G.** RNA Immunoprecipitation (RIP) with anti-PKM2 followed by RT-PCR with H19 mRNA in Hep3B transfected with pCMV-miR, pCMV-miR675, pCMV-miR675 plus pcDNA3.1-HP1α. IgG IP as negative control. H19 cDNA sequence as INPUT. **H.** Western blotting analysis for PKM2 and its polymer in Hep3B transfected with pCI--H19. β-actin as internal control. **I.** Western blotting analysis for C-Myc, Pim1, H-Ras, CDK4, CyclinD1, RB and PCNA analysis in Hep3B transfected with pCI-H19. β-actin as internal control. **J.** C-myc, Ras, Pim1, CyclinD1 and pRB promoter luciferase activity assay in Hep3B transfected with pCI-H19 respectively. Each value was presented as mean±standard error of the mean (SEM). **K.** Western blotting analysis for PKM2 and its polymer in Hep3B transfected with pGFP-V-RS, pGFP-V-RS-miR675, pGFP-V-RS-H19, pGFP-V-RS-EGR1, pCMV-miR675 plus pGFP-V-RS-H19, pCMV-miR675 plus pGFP-V-RS-EGR1. β-actin as internal control. L. Western blotting analysis for H-Ras, C-Myc, CyclinD1 in Hep3B transfected with pCMV-mir, pCMV-miR675, pCMV-miR675 plus pGFP-V-RS-H19, pCMV-miR675 plus pGFP-V-RS-PKM2, pCI-H19 plus pGFP-V-RS-PKM2. β-actin as internal control.

Given that pre-miR675 is embedded in H19's first exon, we should consider whether ectopic overexpressed H19 can produce mature miR675. However, as shown in Figure 6Aa, mature miR675 turnout was significantly decreased in Hep3B cells transfected with pCI-H19 compared to control (*P* < 0.01). As shown in Figure 6Ab, mature miR675 turnout was significantly not altered in HepG2 cells transfected with pCI-H19 or pGFP-V-RS-H19 compared to control (*P* < 0.01). It means that H19 may not act as a miR675 precursor. To identify whether miR675 oncogenic activity was caused by H19, we further determine the H19 function in liver cancer cells. In Hep3B cell line transfected with pCI-H19, the H19 expression was significantly enhanced in the Hep3B transfected with pCI-H19 compared to control (Figure 6B). Next, we performed the cell growth assay *in vitro* and colony formation ability test in liver cancer cell line. As shown in Figure 6C, cell proliferation capacity was significantly improved in Hep3B cell line transfected with pCI-H19 compare to control in the second, third and fourth day, respectively (*P* < 0.01). As shown in the Figure 6D, Cell colony formation rate was significantly higher in Hep3B cell line transfected with pCI-H19 than in control (66.9±9.4 % vs 26.9±4.9%, *P* < 0.01). BrdU positive cells rate was significantly higher in H19 overexpressed Hep3B cell line than that in control (47.4±9.5 % vs 23.7±5.8%, *P* < 0.01) (Figure 6E). Importantly, xenograft tumor was greater in H19 overexpressed group than that in control (1.75±0.12 gram vs 0.57±0.19gram, *P* < 0.01) (Figure 6F). Intriguingly, as shown in Figure 6G, the interaplay between PKM2 and H19 mRNA was significantly intensified in Hep3B cell line transfected with pCMV-miR675 compared to control. However, the interplay between PKM2 and H19 mRNA was significantly reduced in Hep3B cell line transfected with pCMV-miR675 plus pcDNA3.1-HP1α compared to control. It may be caused by the reduced H19. Meaningfully, the PKM2 monomer (58KD), PKM2 dimer (116KD), PKM2 tetramer (232KD), PKM2 hexamer (348KD) were significantly increased in Hep3B cell line transfected with pCI-H19 compared to control respectively (Figure 6H). Ultimately, our results showed that C-Myc, Pim1, H-Ras, CDK4, CyclinD1, PCNA were increased and RB was decreased in H19 overexpressed Hep3B cell lines compared to control (Figure 6I). Moreover, C-myc (362168.7±84322.7 vs 64518.3±9667.5, *P* < 0.01), pim1 (952907.3±175384.8 vs 13625.0±26379.8, *P* < 0.01), Ras (429923.7±71421.3 vs 25787.1±4911.64, *P* < 0.01), CyclinD1 (675313.3±98493.1 vs 87377.7±8480.7, *P* < 0.01 ) promoter luciferase activity were significantly increased and RB promoter luciferase activity (7554.3±1329.4 vs 45125.2±6395.7, *P* < 0.01 ) were significantly decreased in H19 overexpressed Hep3B cell lines compared to control (Figure 6J). Further on, the PKM2 monomer (58KD), PKM2 dimer (116KD), PKM2 tetramer (232KD) were significantly decreased in Hep3B cell line transfected with pGFP-V-RS-miR675, pGFP-V-RS-H19, pGFP-V-RS-EGR1, pCMV-miR675 plus pGFP-V-RS-H19, pCMV-miR675 plus pGFP-V-RS-EGR1 compared to control respectively (Figure 6K). On the other hand, our results showed that C-Myc, H-Ras, CyclinD1 were increased Hep3B cell lines transfected with pCMV--miR675 and decreased in Hep3B transfected with pCMV-miR675 plus pGFP-V-RS-H19, pCMV-miR675 plus pGFP-V-RS-PKM2, pCI-H19 plus pGFP-V-RS-PKM2 compared to control respectively (Figure 6L). Taken together, miR675 upregulates H19 that accelerates hepatocarcinogenesis through activating PKM2 to alter oncogenes expression and function positively.

### miR675 oncogenic function depends on activity of PKM2

**Figure 7:**
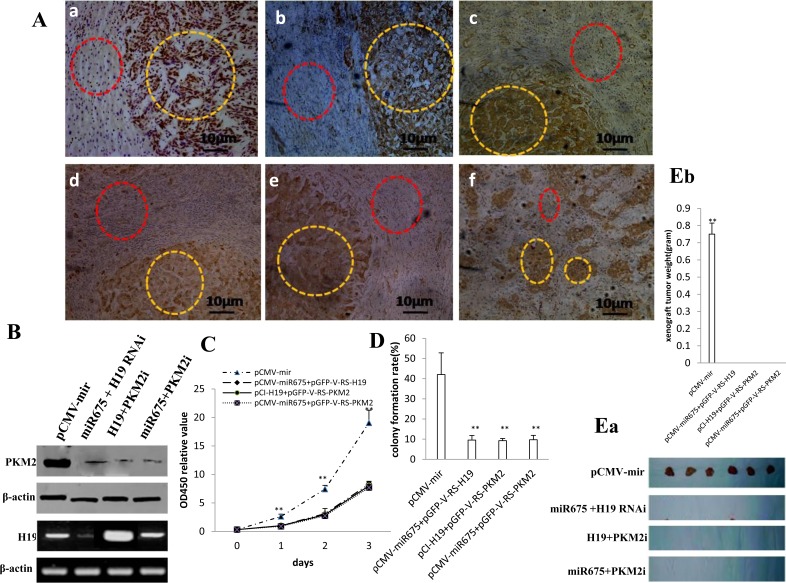
miR675 oncogenic action depends on PKM2 activity **A.** The representative analytic results of in situ hybridization for H19 and miR675, and immunohistochemistry staining for PKM2, HP1α, EGR1 in formalin-fixed, paraffin-embedded human liver cancer tissue (*indicated with yellow Dotted circles*) and their paired adjacent noncancerous tissues (*indicated with Dotted red circles*) from the same patient (DAB stainning, original magnification×100). **B.** Western blotting for PKM2 and RT-PCR analysis for H19 in HepG2 cell lines transfected with pCMV-miR, pCMV-miR675 plus pGFP-V-RS-H19, pCMV-miR675 plus pGFP-V-RS-PKM2, pCCI-H19 plus pGFP-V-RS-PKM2. **C.** Cell proliferation assay was performed in 96-well format using the CCK8 cells proliferation kit to determine the cell viability as described by the manufacturer. Each sample was assayed in triplicates for 3 days consecutively. Cell growth curve was based on the corresponding the relative values of OD450 and each point represents the mean of three independent samples. Data are means of value from three independent experiments, bar±SEM. **, *P* < 0.01 ;*, *P* < 0.05. **D.** Cell plate colony formation ability assay. Data are means of value from three independent experiment, bar±SEM. **, *P* < 0.01 ;*, *P* < 0.05. **E.** a. The photography of xenograft tumors from Balb/C nude mice injected with HepG2 cells transfected with pCMV-mir, pCMV-miR675 plus pGFP-V-RS-H19, pCMV-miR675 plus pGFP-V-RS-PKM2, pCI-H19 plus pGFP-V-RS-PKM2 subcutaneously at armpit. b. The xenograft tumors weight (*gram*) in two groups indicated in *left*. Data were means of value from six Balb/C nude mice, mean±SEM, n = 6, *, *P* < 0.05;**, *P* < 0.01.

Given that miR675 activates PKM2 through H19 in liver cancer cell, we should consider whether miR675 oncogenic function depends on activity of PKM2. At the first time, our results showed that the expression level of miR675, H19, HP1α, EGR1, PKM2, H-Ras were consistent in 10 cases of liver cancer patients and their upregulated expression rate added up to 100% (liver cancer tissue *vs* paracancerous liver tissue) (Figure 7A). Next, we analysed the PKM2 and H19 expression in stable HepG2 cell lines. PKM2 expression was decreased in stable HepG2 cell lines transfected with pCMV-miR675 plus pGFP-V-RS-H19, pCMV-miR675 plus pGFP-V-RS-PKM2, pCI-H19 plus pGFP-V-RS-PKM2 compared to pCMV-miR control. H19 expression was decreased in stable Hep3B cell lines transfected with pCMV-miR675 plus pGFP-V-RS-H19 and increased in stable HepG2 cell line pCI-H19 plus pGFP-V-RS-PKM2 (Figure 7B). Cell proliferation ability was significantly reduced in stable HepG2 cell lines transfected with pCMV-miR675 plus pGFP-V-RS-H19, pCMV-miR675 plus pGFP-V-RS-PKM2, pCI-H19 plus pGFP-V-RS-PKM2 compared to pCMV-miR control (*P* < 0.01) (Figure 7C). Cell colony formation ability was significantly decreased in stable HepG2 cell lines transfected with pCMV-miR675 plus pGFP-V-RS-H19, pCMV-miR675 plus pGFP-V-RS-PKM2, pCI-H19 plus pGFP-V-RS-PKM2 compared to pCMV-miR control (9.55±2.25%, 9.26±1.06%, 9.72±2.17% vs 42.04±10.77%, *P* < 0.01) (Figure 7D). Strikingly, xenograft tumors were produced only in stable HepG2 cell lines transfected pCMV-miR control (Figure 7Ea) and the xenograft average weight added up to 0.75±0.064 gram (*n* = 6, *P* < 0.01) (Figure 7Eb). Obviously, once PKM2 activity was lost, the tumorigenesis ability of miR675 was caused. Together, these observations suggest PKM2 determines the miR675 oncogenic action partly, at least in the human liver cancer cells.

## DISCUSSION

**Figure 8:**
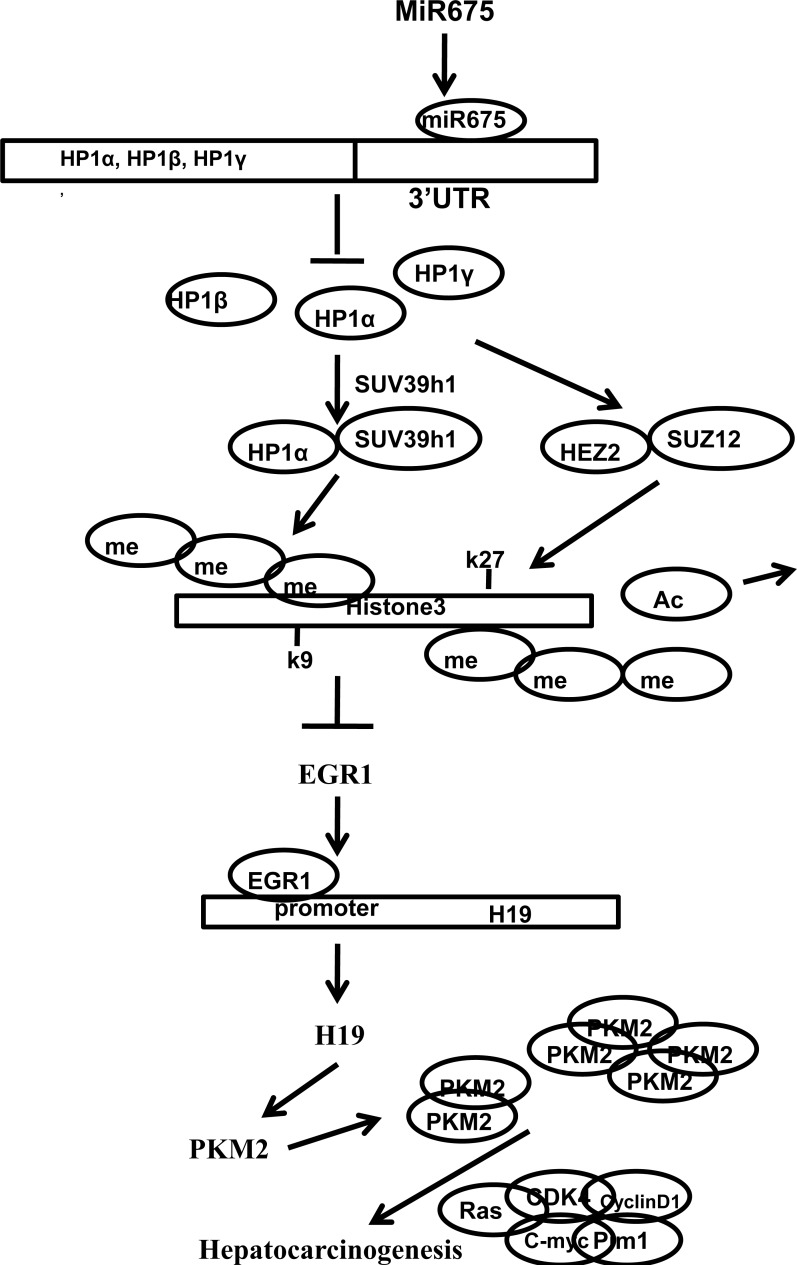
The schematic illustrates a model that miR675 is involved in the epigenetic regulation of H3K9me3, H3K27me3 for gene expression in tumorigenesis miR675 overexpression promotes and silencing miR675 attenuated liver cancer cell growth *in vitro* and *in vivo*. Mechanistically, miR675 decreases the heterochromatin protein HP1α, HP1β, HP1γ expression in human liver cancer cells which causes a marginal decrease of the total histone H3 lysine 9 trimethylation (H3K9me3) and total histone H3 lysine 27 trimethylation (H3K27me3) and a increase of total histone H3 lysine 27 acetylation (H3K27Ac). Notably, a significant reduction of H3K9me3 and H3K27me3 loading onto the promoter region of EGR1 which triggers EGR1 transcription, sumoylation and activation to upregulate H19, a large intergenic non-coding RNA (lincRNA). Intriguingly, H19 induces and activates tumor-specific pyruvate kinase M2 (PKM2) which is essential for the warburg effect in its dimer and for gene expression in its teramer during tumorigenesis.

It is well known that microRNAs (miRNAs) are short non-coding RNAs that are involved in post-transcriptional regulation of gene expression in multicellular organisms by affecting both the stability and translation of mRNAs. Our studies are now indicated to evaluate the effects of miR675 in liver cancer (Figure 8). Our present findings clearly demonstrate that miR675 overexpression promotes and silencing miR675 attenuated liver cancer cell growth *in vitro* and *in vivo*. To our knowledge, this is the first report demonstrating miR675 plays a positive role in liver carcinogenesis through the cascade of miR675-HP1α-EGR1-H19-PKM2 signaling. To this data, we report that the upregulated expression level of miR675, H19, HP1α, EGR1, PKM2, H-Ras were consistent in liver cancer patients and miR-675 upregulates long noncoding RNA H19 through activating EGR1 in human liver cancer cells. Strikingly, we confirm how miR675-HP1α-EGR1-H19-PKM2 cascade might be played an important role in hepatocarcinogenesis. On the other hand, we also proposed a key role for the miR-675 in upregulation of H19 that may induce and activate PKM2, in turn, responsible for changes in gene expression relevant in hepatocarcinogenesis (e.g. C-myc, Pim1, Ras, CyclinD1, RB1). Obviously, this is a new linkage of miR-675-HP1α-ERG1-H19-PKM2 in human liver cancer.

It is worth mentioning that miR675 play an important role in the occurrence of hepatocellular carcinoma. In this report, we focused mainly on the view that miR675 promotes human hepatocarcinogenesis by activating PKM2 dependent on the reduction of HP1 isoforms and the increase of H19. To date, accumulating evidence indicates that miR675 plays a critical role in cancer development and miR675 possess a strong carcinogen properties. Actually, our observations are consistent with these previous reports. For examples, miR-675 modulates human gastric cancer cell proliferation by targeting tumor suppressor Runt Domain Transcription Factor1 (RUNX1) [50];miR-675 was found to be upregulated in human colorectal cancer (CRC) tissues and the tumor suppressor retinoblastoma (RB) was confirmed to be a direct target of miR-675 [51]. Overexpression of the miR-675 in hepatocellular carcinoma links a dramatic upregulation of proliferative and growth capacity [52]. Herein, our results showed that miR675 promotes hepatocarcinogenesis and progress through miR675-HP1α-EGR1-H19-PKM2 cascade signaling pathway. The involvement of miR675 promotion of liver cancer cell growth is supported by results from four parallel sets of experiments: (1) The upregulated expression level of miR675, H19, HP1α, EGR1, PKM2, H-Ras were consistent in liver cancer patients ; miR675 promotes the liver cancer cells malignant proliferation *in vitro* and accelerates liver cancer growth *in vivo*. (2) Mechanistically, miR675 inhibits the heterochromatin1 isoforms (HP1α, HP1β, HP1γ) expression in human liver cancer cells which causes a dramatically decrease of the total histone H3 lysine 9 trimethylation (H3K9me3), histone H3 lysine 27 trimethylation (H3K27me3) and a increase of histone H3 lysine 27 acetylation (H3K27Ac); (3) Next, a significant reduction of the H3K9me3 and H3K27me3 and the increment of H3K27Ac occupancy on the promoter region of EGR1 triggers EGR1 transcription, sumoylation and activation which upregulates lincRNA H19; (4) Ultimately, H19 may induce and activate tumor-specific pyruvate kinase M2 (PKM2) which is essential for the warburg effect in its dimer and for gene expression in its teramer during tumorigenesis. Evidently, miR675 is crucial for cell growth and viability in liver cancer cells. According to the aforementioned findings and reports, it is thus clear that miR675 has a strong carcinogenic ability.

It has been confirmed that heterochromatin protein 1 is a conserved eukaryotic chromosomal protein that is prominently associated with pericentric heterochromatin and mediates the concomitant gene silencing. Mechanistic studies implicate HP1 family proteins as ‘hub proteins,’ able to interact with a variety of chromosomal proteins through the chromo-shadow domain (CSD), as well as to recognize key histone modification sites [primarily histone H3 di/trimethyl Lys9 (H3K9me2/3)] through the chromodomain (CD). HP1 function is altered by context, and potentially by post-translational modifications [53, 54]. For example, HP1α interacts with the Suz12 subunit of the polycomb repressive complex 2 (PRC2) [55] and H3 tail cleavage could serve to release polycomb proteins from K27 methylated H3. A conformational switch in HP1 releases auto-inhibition to drive heterochromatin assembly heterochromatin protein 1 (HP1), which recognizes H3K9-methylated chromatin, oligomerizes and forms a versatile platform that participates in diverse nuclear functions, ranging from gene silencing to chromosome segregation [56]. In this report, we identify heterochromatin protein 1 isoforms are valid targets of miR675 in liver cancer. Our data suggest that miR675 reduced the HP1α expression on the transcriptional level through targeting for HP1α 3′ untranslational region. In particular, we also proved miR675 altered the epigenetic modifications on histone through HP1α reduction. Further on, it lead to increase the early growth response gene1 (EGR1) turnout through transcriptional regulation based on reducing the complex output of HP1α-SUV39H1-Histone3, HP1α-SUV39H1-H3K27Ac, HP1α-SUZ12-EZH2 which caused the dramatically reduction of H3K9me3, H3K27me3 and the increment of H3K27Ac. Strikingly, it promoted the EGR1 occupancy on H19 imprinting control region (ICR) which ultimately enhances H19 expression, suggesting that HP1 isoforms HP1α acts as the hub of miR675 epigenetic regulation function. In addition, our results showed that H19 promoter activity and H19 expression were highly enhanced in cells coexpressing with miR-675 and EGR1 expression when compared with cells expressing EGR1. It suggest EGR1 and miR-675 may function on H19 promoter in different pathways. On the other hand, It also suggest EGR1 can enhance miR675 function that triggers H19 transcription.

There is plenty of evidence that H19 is a oncogenic long noncoding RNA. For example, the levels of H19 was overexpressed in pancreatic ductal adenocarcinoma (PDAC) [57]. H19 was shown to be regulated by c-Myc in Bcr-Abl-expressing cells. [58]. H19 levels are remarkably increased in bladder cancer tissues, and upregulated H19 enhances bladder cancer metastasis by associating with EZH2 and inhibiting E-cad expression [59]. Epigenetic activation of the MiR-200 family contributes to H19-mediated metastasis suppression in hepatocellular carcinoma [60]. Polyploidization of murine mesenchymal cells is associated with suppression of the long noncoding RNA H19 and reduced tumorigenicity [61]. Up-regulated long non-coding RNA H19 contributes to proliferation of gastric cancer cells [62]. Although increment of H19 may partly contribute to miR675 medicated promotion of liver cancer cell growth, our findings in this study provide novel evidence for an active role of H19 in miR675-mediated promotion of liver cancer cell growth. This assertion is based on several observations: (1) miR675 enhanced the H19 transcription although ectopic H19 may not produce the mature miR675. (2) H19 can accelerate hepatocarcinogenesis through activating PKM2 to alter oncogenes expression and function, e.g. C-myc, H-ras, pim1, CDK4, CyclinD1, RB. (3) miR675 oncogenic function will be abrogated if H19 is knocked down in liver cancer cells. These findings are noteworthy that H19 decides in miR675 oncogenic action through mediating various biological processes including cell proliferation, differentiation. It is very clear that H19 is connection of miR675-PKM2 axis that may contribute to hepatocarcinogenesis. It follows that H19 is a manipulator for miR675′s oncogenic activity.

It is worth noting that PKM2 activity alteration is absorbing and of great concern. We observed that miR675 increased PKM2 expression and its activity through H19. Many cancer cells have increased rates of aerobic glycolysis, a phenomenon termed the Warburg effect. PKM2 expression was shown to be necessary for aerobic glycolysis and to provide a growth advantage to tumors. Knockdown of pyruvate kinase in tumor cells leads to a decrease in the levels of pyruvate kinase activity and an increase in the pyruvate kinase substrate phosphoenolpyruvate. PKM2 is dispensable for tumor maintenance and growth *in vivo,* suggesting that other metabolic pathways bypass its function [63]. PKM2 was frequently over-expressed in human HCC and its over-expression was associated with aggressive clinicopathological features and poor prognosis of HCC patients. Furthermore, knockdown of PKM2 suppressed aerobic glycolysis and cell proliferation in HCC cell lines *in vitro*. Importantly, knockdown of PKM2 hampered HCC growth in both subcutaneous injection and orthotopic liver implantation models, and reduced lung metastasis *in vivo* [64]. Strikingly, PKM2 directly binds to histone H3 and phosphorylates histone H3 at T11 upon EGF receptor activation. PKM2 as a protein kinase in its nonmetabolic functions of histone modification is essential for its epigenetic regulation of gene expression and tumorigenesis [65]. PKM2 is upregulated in multiple cancer types and contributes to the Warburg effect by unclear mechanisms. Nuclear PKM2 acts as a coactivator of β-catenin to induce c-Myc expression, resulting in the upregulation of GLUT1, LDHA and, in a positive feedback loop [66]. It is evident that activation of PKM2 may play an important role in miR675 oncogenic action in liver cancer. Our findings in this study provide novel evidence for an active role of PKM2 in miR675-mediated promotion of liver cancer cell growth. This assertion is based on several observations: (1) miR675 upregulates H19, in turn, H19 increased the M2 isoform of pyruvate kinase (PKM2) expression and the formation of the PKM2 monomer, PKM2 dimer, PKM2 tetramer, PKM2 hexamer. (2) H19 and miR675 may activate PKM2. (3) Once PKM2 activity was lost, the tumorigenesis ability of H19 and miR675 was caused. PKM2 determines the miR675 and H19 oncogenic action partly, at least in liver cancer. It is suggest that miR675 and H19 tumorigenic action may require PKM2 participation. That is to say that PKM2 determines the carcinogenic effect of miR675 and H19. In view of this reason, we infer that miR675 and H19 may lead to PKM2 phosphorylation alternation, making PKM2 produce multimers.

In conclusion, we first proved that miR675 exerts its effect in part through the upregulation of H19 and PKM2 expression. Our present approaches provided an unequirocal evidence for critical oncogenic roles of the miR675 in hepaocarcinoma and supported the notion that miR675 may be an alternative bona fide promoting factor of hepatocarcinoma. We presented three miR675 novel mechanisms. Firstly, miR675 inhibits the HP1 isoforms expression and activates EGR1; Secondary, miR675 triggers EGR1 occupancy on *H19* promoter region and enhances H19 transcription positively; Thirds, miR675 controls PKM2 polymer formation dependent on H19 upregulation. On the basis of these mechanisms, miR675 exerts a tumorigenic functions through miR675-HP1α-EGR1-H19-PKM2 cascade signaling pathway in liver cancer. Although miR675 ‘s oncogenic function was due to decrease the HP1 isoforms and increase H19, PKM2 in liver cancer cells, we further confirm how miR675-HP1-EGR1-H19-PKM2 axis might be played an important role in hepatocarcinogenesis and progression. However, we have fully not understood the accuracy mechanism of miR675, such as, how miR675 controls HP1 isoforms dynamics change ? How miR675 regulates PKM2 polymer formation and drives the PKM2 from cytoplasmic yo nuclear? What are the recruitment factors, partners of miR675 during genes regulation and control? What is the clinic significance of miR675? etc. In this report, we focused mainly on the view that miR675 promotes human hepatocarcinogenesis by activating H19 dependent on reduction of HP1 isoforms. Our present findings open the possibility that targeting miR675 might prove to be an alternative therapeutic strategy, e.g. Baculovirus-mediated miRNA regulation to suppress hepatocellular carcinoma tumorigenicity and metastasis [67]. It will produce an important implication for treatment and diagnosis of hepatocarcinoma. It is worth paying attention that we confirm that deciphering the molecular basis of miR675 in hepatocarcinogenesis is very important for us to apply miR675 in clinic diagnosis and therapy later.

## MATERIAL AND METHODS

### Cell lines and plasmid

Human hepatoma cell lines Hep3B and HepG2 were obtained from the Cell Bank of Chinese Academy of Sciences (Shanghai, China). These cell lines were maintained in Dulbecco's modified Eagle medium (Gibco BRL Life Technologies) supplemented with 10% fetal bovine serum (sigma) in a humidified atmosphere of 5% CO_2_ incubator at 37°C. pCMV-miR, pCMV-miR675 (MI0005416), pGFP-V-RS, pCMV-AC-GFP, pCMV6-entry-EGR1, pGFP-V-RS-EGR1, pMitTarget were purchased from Origene (Rockville, MD, USA) and pcDNA3.1, pcDNA3.1-HA--HP1α, pcDNA3.1-HA--HP1β, pcDNA3.1-HA--HP1γ, pBS-H19, pGL3-C-myc, were purchased from Addgene (Cambridge MA, USA). pCI-H19, pMirTarget-HP1α, β, γ3′UTR, pMirTarget-mutant HP1α, β, γ3′UTR, pCMV-mutant miR675, pGFP-V-RS-mir675, pGFP-V-RS-H19, pGFP-V-RS-PKM2, pGL3-EGR1, pGL3-H-Ras, pGL3-CyclinD1, pGL3-Pim1, pGL3-RB was constructed by ourselves.

### Cell transfection and stable cell lines

Hep3B or HepG2 cells were transfected using transfection reagent lipofectamine^R^ 2000 (Invitrogen) according to manufacturer's instructions respectively. For screening miR675 overexpression or knockdown Hep3B or HepG2 stable cell lines, these cells were plated in the selective medium containing 2500 μg/ml G418 (Invitrogen) or 1μg/ml Puromycin (Calbiochem) in forty-eight hours after transfection. For the next 4-8 weeks, the selective media were replaced every 3 days. Once the distinct conoly of surviving cells were transferred into 96-well plate and continued to maintain cultures in selected media. Transfection efficiency was observed by GFP imaging and measured by real-time RT-PCR, Western blotting.

### Reverse-transcriptase polymerase chain reation

Total RNA was purified using Trizol (Invitrogen) according to manufacturer's instructions. cDNA was prepared by using oligonucleotide (dT)_18_ and a SuperScript First-Strand Synthesis System (Invitrogen). The PCR amplification kit (TaKaRa) were adopted according to the manufacturer's instructions. H19 cDNA was amplified using the upstream primer (5′-attgcgcagcaaggaggctg-3′) and the down stream primer (5′-cctccctcctgagagctcat-3′) (synthesized by Shenggong, Shanghai, China) under the PCR reaction conditions performed in 35 cycles with each cycle consisting of a denaturation step (94°C for 30 seconds, and 3 minutes for the first cycle only), an annealing step (58°C for 30 seconds) and an elongation step (72°C for 30 seconds, 10 minutes for the last cycle only ).β-actin served as a internal control for the efficiency of the RT-PCR. β-actin primer: P1:5′-gggaaatcgtgcgtgacatt-3′;P2:5′-ctcaggaggagcaatgatct-3′;Real-time PCRβ-actin primer: P1:5′-ggtcatcaccattggcaatg-3′;P2:5′-aaggtagtttcgtggatgcc-3′. PCR products were analyzed by 1.0% agarose gel electrophoresis and visualized by ethidium bromide staining using Image imaging system (Baygene).

### MicroRNA detection

Total RNA was isolated from cultured cells using Trizol (Invitrogen, Carlsbad, CA, USA) according to the manufacturer's protocol. Real-time RT-PCR-based detection of mature miR-675 and U6 snRNA was achieved with the miRNA Detection kit (including a universe primer, U6 primers, Qiagen) and miR675 specific upsteam primers (Origene, USA). qRT-PCR was performed with a StepOnePlus real-time PCR system (Applied Biosystems), a SuperScript First-Strand Synthesis System (Invitrogen, Carlsbad, CA, USA) and Power SYBR Green PCR Master Mix (Applied Biosystems) in accordance with the manufacturers' protocols. Each sample was run in triplicate. *C*_t_ values for miR675 were calculated and normalized to *C*_t_ values for U6 snRNA. The following primers were used: human mi675P1: 5′-TGGTGCGGAGAGGGC-3′ and P2:5′-GAACATGTCTGCGTATCTC-3′. U6 primer:P1:5′-GCTTCGGCAGCACATATACT-3′;P2:5′-GGAACGCTTCACGAATTTGC-3′

### Western blotting

The logarithmically growing cells were washed twice with ice-cold phosphate-buffered saline (PBS, Hyclone Lab. INC) and lysed in RIRP lysis buffer [50 mM Tris-HCl (pH 7.4), 150 mM NaCl, 1% NP-40, 0.1% SDS] containing protease inhibitor cocktails (Roch, Diagnostics, Indianapolis IN, USA). Cells lysates were centrifuged at 12, 000g for 20 minutes at 4°C after sonication on ice, and supernatants were separated. After being boiled for 10 minutes in the presence of 2-mercaptoethanol, samples containing cells or tissue lysate proteins were separated on a 10% sodium dodecyl sulfate-polyacrylamide gel electrophoresis (SDS-PAGE) and transferred onto a nitrocellulose membranes Membranes were stained and then blocked in 10% dry milk-TBST [20mM Tris-HCl (PH 7.5)], 0.1% Tween 20) for 1 h at 37°C. Following three washes in Tris-HCl pH 7.5 with 0.1% Tween 20, the blots were incubated with antibody (appropriate dilution) overnight at 4°C. Following three washes, membranes were then incubated with secondary antibody for 60 min at 37°C or 4°C overnight in TBST. Signals were visualized by ODYSSEY infrared imaging system (LI-COR). Standard western immunoblotting procedures were used with the following antibodies: Rabbit polyclonal anti-HP1α, Rabbit polyclonal anti-HP1β, Rabbit polyclonal anti-HP1γ, Rabbit polyclonal anti-H3K9me3, Rabbit polyclonal anti-H3K27me3, Rabbit polyclonal anti-SUV39h1, Rabbit polyclonal anti-Histone3, Rabbit polyclonal anti-pHistone3, Rabbit polyclonal anti-H3K4me3, Rabbit polyclonal anti-H3K27Ac, Rabbit polyclonal anti-PKM2, Rabbit polyclonal anti-Pim1, mouse monoclonal anti-EGR1, mouse monoclonal anti-SUZ12, mouse monoclonal anti-EZH2, mouse monoclonal anti-C-Myc, mouse monoclonal anti-Ras, mouse monoclonal anti-CDK4, mouse monoclonal anti-CyclinD1, mouse monoclonal anti-RB, mouse monoclonal anti-PCNA, mouse monoclonal anti-β-actin were purchased from Santa Cruz, Biotech. IRDye 680LT /IRDye 800CW secondary antibodies were purchased from LI-COR scientific company. All other reagents and compounds were analytical grades (Sigma, Promega, Shengong, etc).

### Co-immunoprecipitation (IP)

Cells were lysed in RIRP lysis buffer containing protease inhibitor cocktails (Roch, Diagnostics, Indianapolis IN, USA). Five-hundred-microliter cell lysates was used in immunoprecipitation with antibody. In brief, protein was pre-cleared with 30μl protein G/A-plus agarose beads (Santa Cruz, Biotechnology, Inc. CA) for 1 hour at 4°C and the supernatant was obtained after centrifugation (5, 000rpm) at 4°C. Precleared homogenates (supernatant) were incubated with 2 μg of antibody and/or normal mouse/rabbit IgG by rotation for 4 hours at 4°C, and then the immunoprecipitates were incubated with 30μl protein G/A-plus agarose beads by rotation overnight at 4°C, and then centrifuged at 5000rpm for 5 min at 4°C. The precipitates were washed five times×10min with beads wash solution (50 mM pH7.6 TrisCl, 150mMNaCl, 0.1%NP-40, 1mM EDTA) and then resuspended in 40μl 2×SDS-PAGE sample loading buffer to incubate for 10 min at 100°C. Then Western blot was performed with a another related antibodies.

### DNA pull down

Cells were lysed by sonication in HKMG buffer (10 mM HEPES, PH7.9, 100 mM KCl, 5 mM MgCl_2_, 100% glycerol, 1 mM DTT, and 0.5% NP40) containing protease inhibitors for the preparation of nuclear exact. Equal amount of cell nuclear extracts were precleared with Streptavidin-agarose Resin (Thermo) for 1 hours, and then were incubated with 1μg biotinylated EGR1 site double-stranded-oligonucleotides (forward:5′-biotin-cgcccccgccgcccccgc-3′and reverse: 5′-biotin-gcgggggcggcgggggcg-3′) and together with 10μg poly (dI-dC) at 4°C for 24 hours. DNA-bound proteins were collected with the incubation with streptavidin-agarose Resin for 1 hour with gently shaking to prevent precipitation in solution. Following five times washings of the resin bound complex with 0.5-1.0 ml of binding buffer, the samples were boiled and subjected to SDS-PAGE and Western blotting analysis.

### Dual luciferase reporter assay

Cells (1×10^5^/well of a six-well plate) were transiently transfected with 1.5 μg of luciferase construct (pMirtarget-HP1αβγ3′UTR, pGL3-EGR1, pBS-H19, et al) or indicated plasmids and 0.2 μg of pRL-tk (promega) with the use of the Lipofectiamine^TM^ 2000 (Invitrogen). Transfection was performed with at least three different batches of each reporter plasmid. After incubation for 36-48 hours, the cells were harvested with Passive Lysis Buffer (Promega), and luciferase activities of cell extracts were measured with the use of the Dual luciferase assay system (Promega) according to manufacturer's instructions. luciferase activity was measured and normalized for transfection efficiency with Renilla luciferase activity. Transfection was performed with at least three different batches of each reporter plasmid.

### Chromatin immunoprecipitation (CHIP)

Cells were cross-linked with 1% (v/v) formaldehyde for 10 min at room temperature and stopped with 125 mm glycine for 5 min. Crossed-linked cells were washed with phosphate-buffered saline, resuspended in lysis buffer, and sonicated for 8-10 min in a SONICS to generate DNA fragments with an average size of 500-1000 bp. Chromatin extracts were diluted 5-fold with dilution buffer, pre-cleared with Protein-A/G-Sepharose beads, and immunoprecipitated with specific antibody on Protein-A/G-Sepharose beads. After washing, elution and de-cross-linking, the ChIP DNA was detected by traditional PCR (36 cycles) on the PCR Detection system (Bio-Rad). The following primer pairs were used: CHIP EGR1 primer EGR1 promoter DNA (AJ243425) :P1: P1:5′-cagcaccttatttggagtgg-3′ (2011-2030); P2:5′-acctccatcctgcagggtag-3′ (2191-2210). CHIP EGR1 negative primer :P1:5′-aagctactcgagaggaggag-3′ (183-202); P2:5′-agatggccttgtgtctgaat-3′ (361-380) and H19/promoter: (P1:5′-tgtatttctggaggcttccc-3′;P2: 5′-tcagacacgtagcccgatat-3′).

### RNA immunoprecipitation (RIP)

Cells were lysed (15 min, 0°C) in 100 mM KCl, 5 mM MgCl_2_, 10 mM HEPES [pH 7.0], 0.5% NP40, 1 mM DTT, 100 units/ml RNase OUT (Invitrogen), 400 μM vanadyl-ribonucleoside complex and protease inhibitors (Roche), clarified and stored on at −80°C. Ribonucleoprotein particle-enriched lysates were incubated with protein A/G-plus agarose beads (Santa Cruz, Biotechnology, Inc. CA) together with anti-PKM2 or normal mouse IgG for 4 hours at 4°C. Beads were subsequently washed four times with 50 mM Tris-HCl (pH 7.0), 150 mM NaCl, 1 mM MgCl_2_, and 0.05% NP-40, and twice after addition of 1M Urea. Immunoprecipitates (IPs) were digested with proteinase K (55°C; 30′) and mRNAs were then isolated and purified. RT-PCR was performed with the primers as follows: H19/P1:5′-attgcgcagcaaggaggctg-3′, H19/P2:5′-cctccctcctgagagctcat-3′.

### Cells proliferation assay

Cells at a concentration 4×10^3^ were seeded into 96-well culture plates in 100μl culture medium containing 10% fetal calf serum (FCS). Before detected, add 10μg/well cell proliferation reagent CCK8 (Yeasen) and incubate for 4 hours at 37°C and 5% CO_2_. Measure the absorbance of the samples against a background control as blank using SpectraMax M5 (Molecular Devices, MD, USA) according to the manufacturer instruction. Each sample was assayed in triplicates at daily intervals after seeding for up to 3 days consecutively. Cell growth curve was based on the corresponding the normalized values of OD450 and each point represents the mean of three independent samples.

### Colony-formation efficiency assay

1 × 10^3^ cells were plated on a 10 cm dish, the 10 ml DMEM containing 10%FBS was added into each 10cm dish of the three replicate. Then these dishes were incubated at 37°C in humidified incubator for 10 days. Cell colonies on the dishes were stained with 1 ml of 0. 5% Crystal Violet for more than 1 hour and the colonies were counted.

### BrdU staining

80% confluent cells were cultured for 24 hour before treatment with 10μl BrdU (Roche) for 4 hours. Immunofluorescent staining with an anti-BrdU antibody was performed according to the manufacturer's instructions (Becton Dickinson). BrdU positive cells from ten random chosen fields of at least three independent samples were counted.

### Immunohistochemistry

Tissues were fixed with 4% paraformaldehyde, dehydrated, embedded in paraffin and sectioned at 4μm. Sections were immunohistochemically stained using mouse anti-human monoclonal anti-PKM2, anti-H-Ras, anti-EGR1, anti-HP1α, (Santa Cruz, Biotech). As the secondary antibody, anti-mouse IgG (Horseradish peroxidase linked whole antibody from sheep, GE Healthcare Limited) was used at 100×dilution. Staining was performed using 3, 3-diaminobenzidine (DAB) substrate kit for peroxidase according to the manufacturer's instructions (Vector Laboratories Inc) and counterstained with hematoxylin.

### *In situ* hybridization

For tissus slides, deparaffinization and antigen retrieval (Digest with 20 μg/ml proteinase K in pre-warmed 50 mM Tris for 10 to 20 min at 37°C.). Rinse slides 5 times in distilled water. Immerse slides in ice cold 20% (v/v) acetic acid for 20 sec. Dehydrate the slides by washing for approximately 1 min each wash in 70% ethanol, 95% ethanol and 100% ethanol then air dry. Add 100 μl of hybridization solution to each slide. Incubate the slides for 1 hr in a humidified hybridization chamber at the 42°C. Under heat at 95°C for 2 min, to denature the DIG (Digoxigenin) labeled DNA probe. Drain off the hybridization solution. Add 50 μl of diluted probe per section. Incubate in the humidified hybridization chamber at 42 overnight. While incubating, the sample on the slide can be covered with a cover slip to prevent evaporation. Stringency washes: Wash 1: 50% formamide / 2 × SSC (3 × for 5 min, 37-45°C). Wash 2: 0.1-2 × SSC3 × for 5 min, 25°C to 75°C. Wash twice in MABT (maleic acid buffer containing Tween 20) for 30 min at room temperature. Dry the slides. Transfer to a humidified chamber and add 200 μl blocking buffer to each section (MABT + 2% BSA, milk or serum). Block for 1 to 2 hours, at room temperature. Drain off the blocking buffer. Add the anti-DIG antibody at the required dilution in blocking buffer. Wash slides 5 times with MABT, 10 min for each wash, at room temperature. SABC-DAB staining and washing slides in distilled water. Air dry the slides for around 30 min. Wash in 100% ethanol, then air dry thoroughly. Mount using DePeX mounting solution.

### Xenograft transplantation *in vivo*

Four-weeks male athymic Balb/C mice were purchased from Shi Laike company (Shanghi, China) and maintained in the Tongji animal facilities approved by the China Association for accreditation of laboratory animal care. The athymic Balb/C mice were injected at the armpit area subcutaneously with suspension of 7×10^6^ transfected Hep3B cells or HepG2 cells in 100μl of phosphate buffered saline. The mice were observed four weeks, and then sacrificed to recover the tumors. The wet weight of each tumor was determined for each mouse. A portion of each tumor was fixed in 4% paraformaldehyde and embedded in paraffin for histological hematoxylin-eosin (HE) staining and anti-PCNA immunohistochemical staining. The use of mice for this work was reviewed and approved by the institutional animal care and use committee in accordance with China national institutes of health guidelines.

### Statistical analysis

The significant differences between mean values obtained from at least three independent experiments. Each value was presented as mean±standard error of the mean (SEM) unless otherwise noted, with a minimum of three replicates. The results were evaluated by SPSS20.0 statistical soft (SPSS Inc Chicago, IL) and Student's t-test, Chi-square test were used for comparisons, with *P* < 0.05 considered significant.
